# An LED-Based structured illumination microscope using a digital micromirror device and GPU accelerated image reconstruction

**DOI:** 10.1371/journal.pone.0273990

**Published:** 2022-09-09

**Authors:** Musa Aydın, Yiğit Uysallı, Ekin Özgönül, Berna Morova, Fatmanur Tiryaki, Elif Nur Firat-Karalar, Buket Doğan, Alper Kiraz

**Affiliations:** 1 Department of Computer Engineering, Fatih Sultan Mehmet Vakif University, Istanbul, Turkey; 2 Department of Physics, Koç University, Istanbul, Turkey; 3 KUTTAM, Koç University Research Center for Translational Medicine, Istanbul, Turkey; 4 Department of Molecular Biology and Genetics, Koç University, Istanbul, Turkey; 5 School of Medicine, Koç University, Istanbul, Turkey; 6 Department of Computer Engineering, Marmara University, Istanbul, Turkey; 7 Department of Electrical and Electronics Engineering, Koç University, Istanbul, Turkey; University of Houston, UNITED STATES

## Abstract

When combined with computational approaches, fluorescence imaging becomes one of the most powerful tools in biomedical research. It is possible to achieve resolution figures beyond the diffraction limit, and improve the performance and flexibility of high-resolution imaging systems with techniques such as structured illumination microscopy (SIM) reconstruction. In this study, the hardware and software implementation of an LED-based super-resolution imaging system using SIM employing GPU accelerated parallel image reconstruction is presented. The sample is illuminated with two-dimensional sinusoidal patterns with various orientations and lateral phase shifts generated using a digital micromirror device (DMD). SIM reconstruction is carried out in frequency space using parallel CUDA kernel functions. Furthermore, a general purpose toolbox for the parallel image reconstruction algorithm and an infrastructure that allows all users to perform parallel operations on images without developing any CUDA kernel code is presented. The developed image reconstruction algorithm was run separately on a CPU and a GPU. Two different SIM reconstruction algorithms have been developed for the CPU as mono-thread CPU algorithm and multi-thread OpenMP CPU algorithm. SIM reconstruction of 1024 × 1024 px images was achieved in 1.49 s using GPU computation, indicating an enhancement by ∼28 and ∼20 in computation time when compared with mono-thread CPU computation and multi-thread OpenMP CPU computation, respectively.

## 1 Introduction

Fluorescence microscopy is a key imaging modality enabling visualization of specific sub-cellular structures that are highlighted with fluorescence markers. In wide-field illumination fluorescence microscopy where the sample is illuminated with a homogeneous intensity distribution across the field, the lateral resolution is limited by the diffraction limit ($\frac{\lambda }{2\text{NA}}$) that can be brought down to ∼200 nm at visible wavelengths with the use of high numerical aperture (NA) microscope objectives [1, 2].

In order to achieve resolution improvements beyond the diffraction limit, a number of super-resolution imaging techniques have been developed, i.e. photoactivated localization microscopy (PALM), stochastic optical reconstruction microscopy (STORM), stimulated emission depletion microscopy (STED) and structured illumination microscopy (SIM) [3–7]. In addition to these techniques, the anisotropic resolution of the 2D camera detector can be used to improve the spatial resolution of microscopic images [8]. In this work, SIM is selected as the method of choice due to its relative ease of setup and cost-effective nature. Majority of the SIM setups in the literature employ spatial light modulators (SLMs) [9, 10]. In such an exemplary system, in order to obtain accurate illumination patterns and phase shifts, sequential optical components are used together with an SLM [11]. An SLM positioned in the camera conjugate plane generates the periodic phase patterns. Following the SLM, a liquid crystal waveplate rotates the laser beams to ensure the beam is s-polarized. Finally, a rotating mask is used in order to select the desired diffraction orders. In another work, Markwirth *et al*. developed a real time image reconstruction system using an SLM. In that work, the setup employed a graphics processing unit (GPU) for real time image reconstruction, a Fourier mask and a polarizer were used to select the desired diffraction orders together with an SLM in a SIM configuration [12]. SIM devices developed using an SLM are relatively expensive and fragile [13–15].

A common and cost-effective alternative for illumination pattern generation is the digital micromirror device (DMD) technology. Laser sources have been frequently used with DMDs for generation of high-contrast illumination patterns in SIM setups [16, 17]. Due to its periodic surface, the DMD creates a blazed grating effect and generates multiple diffractive orders. ∓1 diffracted orders are then selected and using a polarizer the equivalence of the polarization of diffracted beams are ensured. Such setups utilizing lasers as sources require high precision alignment and are generally costly. In addition, there are low-cost studies that use LED and DMD instead of laser. Dan *et al*., performed optical sectioning by reconstructing the raw images obtained from the sample illuminated with three DMD illumination patterns with 90° phase difference between them and obtained a 3D high resolution image [18]. In this work, we used a DMD together with LEDs as sources, strongly reducing the complexity of the SIM setup. Hence we took advantage of the low cost, high frame-rate and wide availability of DMD systems [19]. Combined with LED illumination, no additional polarization maintenance was required and diffractive effects were not observed due to the incoherent nature of LED sources. On the other hand, LED illumination is an incoherent illumination mode that reduces speckle noise caused by laser interference [20]. In our setup, square wave modulated intensity patterns were loaded onto the DMD which were later turned into sinusoidally modulated intensity patterns via the diffraction were displayed on the DMD and imaged onto the sample to achieve super-resolution fluorescence microscopy employing conventional striped SIM illumination. Furthermore, the system we have developed has three different colours of programmatically controllable LEDs, enabling multi-spectral imaging of samples with the addition of a suitable filter set.

In this study, we introduce a MATLAB based parallel image reconstruction algorithm utilizing a custom computer code in CUDA programming language that achieves GPU acceleration of SIM reconstruction. Users can run their programs in parallel without having any prior understanding of parallel programming by simply giving arguments to the appropriate routines. Using GPU acceleration we achieve a speed enhancement of up to ∼28 in SIM reconstruction of images with 1028 × 1028 px.

This paper is organized as follows. Section 2 describes the theory of SIM. Section 3 describes the experimental setup. In Section 4, the steps of the image reconstruction algorithm are explained. In Section 5, GPU-based parallel image reconstruction technique is described. In Section 6, the results obtained from this study are shown and discussed. Conclusions are drawn in Section 7.

## 2 Theory of structured illumination microscopy

**Fig 1:**
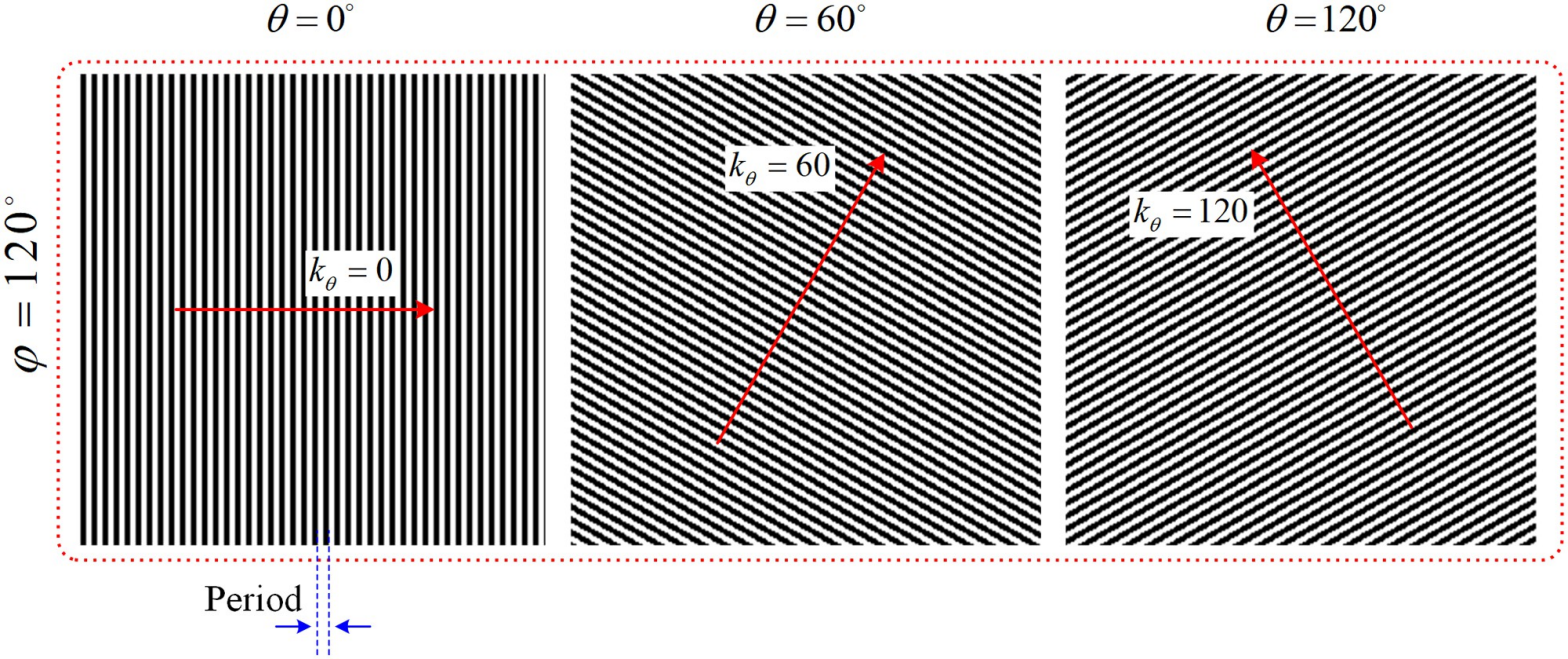
Exemplary illumination patterns created in 3 different orientations *θ*_1_ = 0°, *θ*_2_ = 120°, *θ*_3_ = 60° with the same phase value of *φ* = 120°.

SIM is a microscopic super-resolution imaging modality employing spatially modulated illumination patterns and post-processing for obtaining images with a resolution exceeding the diffraction limit. In conventional SIM, the illumination pattern at the focal plane of the sample consists of a sinusoidal stripe pattern with a high spatial frequency, a well-defined phase and orientation. As an example, Fig 1 shows illumination patterns created in 3 different orientations with the same phase value (the illumination patterns created for all 9 images can be found in S4 Fig in S1 File).

Spatial modulation of the illumination pattern results in shift in the overall frequency spectrum of the image obtained from the sample. This shift enables the detection of high spatial frequency components which are otherwise undetectable due to the diffraction limit set forth by the microscope objective. In SIM, a single high resolution image is created by running the image reconstruction algorithm with the obtained raw images under all illumination patterns.

In order to create the characteristic formulation of a microscopic system, the optical system characteristics can be determined by an impulse response. When an input impulse function is given, the output of the system is calculated by the convolution of the input and the impulse [21]. The basic mathematical formulation of image formation in an optical imaging system is given as [22]:

$$\begin{array}{c} D\left(r\right)=[E_{m}\left(r\right)⊗PSF\left(r\right)], \end{array}$$ (1)

where, *D*(***r***) is the optical intensity information detected by the detector (e.g. CMOS camera), *E*_*m*_(*r*) is the distribution of the sample fluorescence emission, *PSF*(***r***) is the point spread function of the microscope, ***r*** ≡ (*x*, *y*) is two dimensional spatial position vector at the image plane and ⊗ denotes the convolution operation. Fourier transform of Eq 1 reveals:

$$\begin{array}{c} \tilde{D}\left(k\right)=\left[{\tilde{E}}_{m}\left(k\right)\cdot OTF\left(k\right)\right]\;, \end{array}$$ (2)

where, the optical transfer function *OTF*(***k***) is the Fourier transform of *PSF*(***r***) and ***k*** is the spatial frequency vector. *OTF*(***k***) indicates the the spatial frequencies accessible by the microscope. In a conventional fluorescence microscope, the fluorescence intensity detected at the image plane is directly proportional to the illumination light intensity, *I*(***r***), and is calculated as:

$$\begin{array}{c} E_{m}\left(r\right)=I\left(r\right)\cdot S\left(r\right)\;, \end{array}$$ (3)

where *S*(***r***) indicates the fluorescence emission distribution of the sample. Fourier transform of Eq 3 reveals:

$$\begin{array}{c} {\tilde{E}}_{m}\left(k\right)=\tilde{I}\left(k\right)⊗\tilde{S}\left(k\right)\;. \end{array}$$ (4)

Substitution of Eq 4 in Eq 2 reveals:

$$\begin{array}{c} \tilde{D}\left(k\right)=\left[\tilde{I}\left(k\right)⊗\tilde{S}\left(k\right)\right]\cdot OTF\left(k\right)\;. \end{array}$$ (5)

Hence, for uniform illumination, namely the wide-field illumination, *I*(***r***) will take a constant value, and the resulting image will be the product of the sample and the *OTF*(***k***), revealing:

$$\begin{array}{c} \tilde{D}\left(k\right)=\tilde{S}\left(k\right)\cdot OTF\left(k\right)\;. \end{array}$$ (6)

According to Eq 6, all spatial frequency components of the image detected by the microscope will be limited by the spatial frequency bandwidth of the *OTF*(***k***). In SIM however, the sample is illuminated with patterns modulated in the form of sinusoidally distributed stripes. A sinusoidally modulated illumination distribution can be expressed as:

$$\begin{array}{c} I\left(r\right)=I_{0}[1+m\text{cos}(2\pi k_{\theta }\cdot r+\varphi )]\;, \end{array}$$ (7)

where *k*_*θ*_ and *φ* are the magnitude of the frequency vector and initial phase angle value of the sinusoidal illumination pattern, respectively. *I*_0_ and *m* are constants that specify the average light intensity and the modulation depth. Fourier transform of Eq 7 then reveals:

$$\begin{array}{c} \tilde{I}\left(k\right)=I_{0}[\delta \left(k\right)+\frac{m}{2}\cdot e^{i\varphi }\delta \left(k-k_{\theta }\right)+\frac{m}{2}\cdot e^{-i\varphi }\delta \left(k+k_{\theta }\right)]\;. \end{array}$$ (8)

Substituting Eq 8 into Eq 5, the frequency spectrum of the obtained image, which is modulated with sinusoidal illumination pattern, is obtained as:

$$\begin{array}{c} \tilde{D}\left(k\right)=I_{0}\left[\tilde{S}\left(k\right)+\frac{m}{2}e^{i\varphi }\tilde{S}\left(k-k_{\theta }\right)+\frac{m}{2}e^{-i\varphi }\tilde{S}\left(k+k_{\theta }\right)\right]\cdot OTF\left(k\right)\;. \end{array}$$ (9)

Here, the first term, $I_{0}\tilde{S}\left(k\right)$, corresponds to the frequency spectrum obtained by conventional wide-field illumination microscopy. *OTF* imposes a cutoff spatial frequency $k_{c}=2\pi \frac{2NA}{\lambda }$ (*NA* numerical aperture, λ wavelength) such that *k* ≤ *k*_*c*_ is satisfied in the frequency spectrum of the obtained image due to the Abbe diffraction limit [23]. The actual experimental cutoff frequency is generally lower due to additional optical elements and vibrations coupling into the system. Hence, the microscope objective works as a low pass filter in frequency domain, and images can be obtained between ±*k*_*c*_ cutoff bands.

The 2^*nd*^ and 3^*rd*^ terms in Eq 9 ($I_{0}\frac{m}{2}e^{i\phi }\tilde{S}\left(k-k_{\theta }\right)$, $I_{0}\frac{m}{2}e^{-i\phi }\tilde{S}\left(k+k_{\theta }\right)$ respectively) indicate the additional frequency content of the detector image that fall within the cutoff frequency range of *k* ≤ *k*_*c*_ thanks to the sinusoidal modulation of the illumination pattern. This additional information resides outside the *OTF* cutoff frequency and thus can be employed to obtain image resolution beyond the diffraction limit by combining images obtained using sinusoidal illumination patterns with different phases, ***k***_***θ***_ represents the spatial frequency vector of the sinusoidal illumination pattern where *θ* indicates the orientation of the sinusoidal illumination pattern.

In conventional stripe SIM, a total of 9 raw images are obtained. These images are modulated by illumination patterns with three different *θ* values $\left(0,\frac{\pi }{3},\frac{2\pi }{3}\right)$ each generated with three different *φ* values $\left(0,\frac{2\pi }{3},\frac{4\pi }{3}\right)$ at each orientation. As a result of modulation, the center point of the image $\tilde{S}\left(k-k_{\theta }\right)$ is shifted by ***k***_***θ***_. These raw images are then shifted back to their original positions in frequency space as specified in Eq 9. This reveals the following linear set of equations which can be solved for $\tilde{D}\left(k\right)$:

$$\begin{array}{c} {}[\begin{array}{c} {\tilde{D}}_{\varphi_{1}}\left(k\right)\\ {\tilde{D}}_{\varphi_{2}}\left(k\right)\\ {\tilde{D}}_{\varphi_{3}}\left(k\right) \end{array}]=I_{0}M\cdot [\begin{array}{c} \tilde{S}\left(k\right)\cdot OTF\left(k\right)\\ \tilde{S}\left(k-k_{\theta }\right)\cdot OTF\left(k\right)\\ \tilde{S}\left(k+k_{\theta }\right)\cdot OTF\left(k\right) \end{array}]\;, \end{array}$$ (10a)

$$\begin{array}{c} M=[\begin{array}{ccc} 1 & \frac{m}{2}e^{i\varphi_{1}} & \frac{m}{2}e^{-i\varphi_{1}}\\ 1 & \frac{m}{2}e^{i\varphi_{2}} & \frac{m}{2}e^{-i\varphi_{2}}\\ 1 & \frac{m}{2}e^{i\varphi_{3}} & \frac{m}{2}e^{-i\varphi_{3}} \end{array}]\;. \end{array}$$ (10b)

Here, the *OTF* emerges as the most critical parameter needed for solving the set of equations, which is experimentally determined for a given SIM setup. The raw images ${\tilde{D}}_{\varphi_{1}}\left(k\right)$, ${\tilde{D}}_{\varphi_{2}}\left(k\right)$, ${\tilde{D}}_{\varphi_{3}}\left(k\right)$ obtained for three different phase values (*φ*_1_ = 0°, *φ*_2_ = 120°, *φ*_3_ = 240°) are given in Eq 10. Images encoded by illumination patterns with different phase values are obtained when the separated frequency components for the raw images are calculated. $\tilde{S}\left(k\right)$, $\tilde{S}\left(k-k_{\theta }\right)$, $\tilde{S}\left(k+k_{\theta }\right)$ functions are obtained separately when the equation system below is solved:

$$\begin{array}{c} {}[\begin{array}{c} \tilde{S}\left(k\right)\cdot OTF\left(k\right)\\ \tilde{S}\left(k-k_{\theta }\right)\cdot OTF\left(k\right)\\ \tilde{S}\left(k+k_{\theta }\right)\cdot OTF\left(k\right) \end{array}]=M^{-1}[\begin{array}{c} {\tilde{D}}_{\varphi_{1}}\left(k\right)\\ {\tilde{D}}_{\varphi_{2}}\left(k\right)\\ {\tilde{D}}_{\varphi_{3}}\left(k\right) \end{array}]\;. \end{array}$$ (11)

When the modulated raw images are multiplied by the inverse of the *M* matrix, the spectral components are correctly separated according to Eq 11 revealing the solutions for $\tilde{S}\left(k\right)$, $\tilde{S}\left(k-k_{\theta }\right)$, $\tilde{S}\left(k+k_{\theta }\right)$. The resulting nine functions are shifted in the spatial frequency domain, combined, and super resolution images are obtained by inverse Fourier transform. These steps are described in detail in Section 4.

## 3 Experimental setup

**Fig 2:**
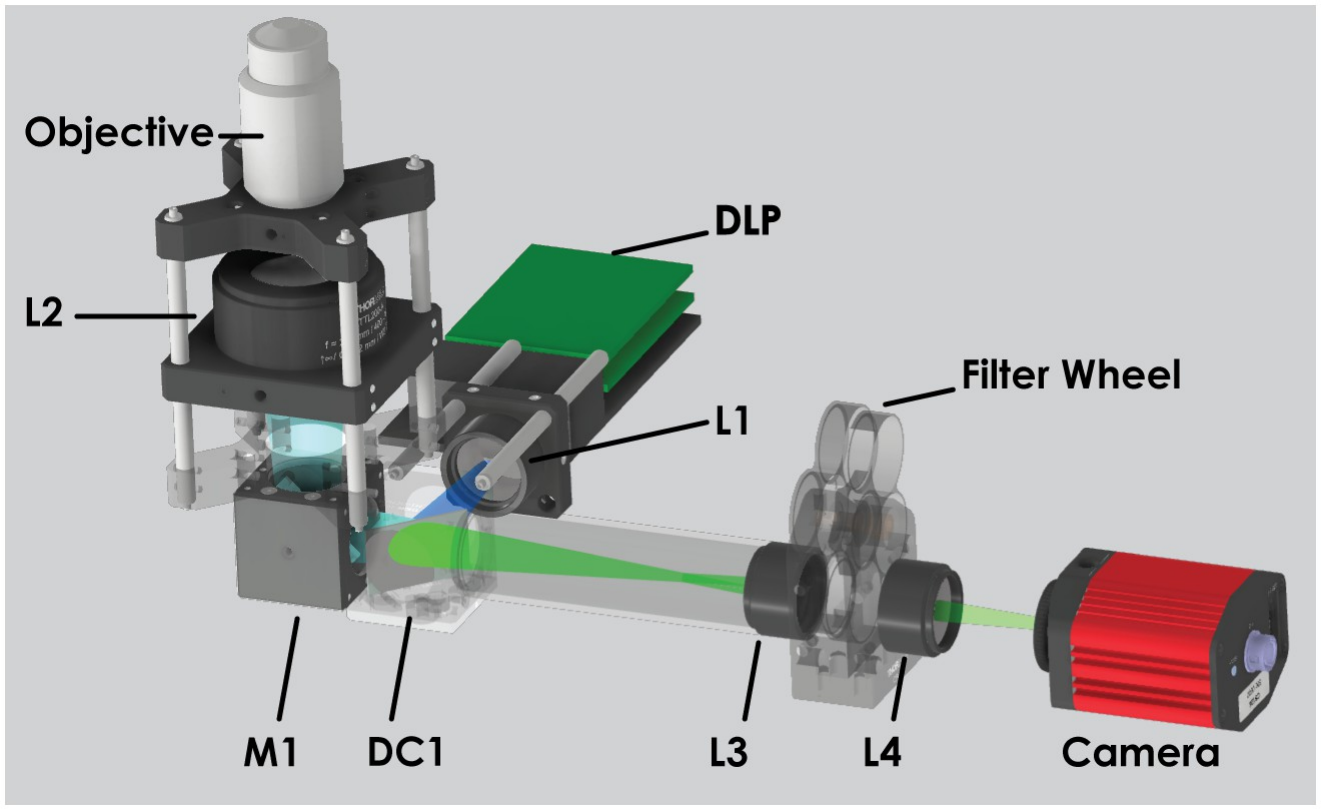
Schematic of the home-built LED-based SIM setup.

A home-built, LED-based, compact, low-cost SIM setup employing a DMD was used in the experiments (Fig 2) (see S16 Fig in S1 File for a picture of the setup).

In the experimental setup, an oil immersion 60x/1.4 N.A. objective and a *f* = 200 mm tube lens (L2) form an infinity corrected microscope with 60*x* magnification. The illumination patterns generated by the DLP projection module (Texas Instruments DLP LightCrafter DLP3000 Evaluation Module) are coupled into the microscope via a *f* = 30 mm lens (L1) and imaged onto the sample in the focal plane of the objective lens.

A dichroic mirror (DC1) and a flip mirror (M1) located in the illumination path are used to reflect the excitation beam onto the sample. The returning signal from the sample is magnified 1.6 times with lenses L2 (*f* = 30 mm and L3 (*f* = 50 mm), and filtered using an appropriate emission filter selected from the wheel. The fluorescence image is registered via a 2MP monochrome sCMOS camera (CS2100M-USB, Thorlabs) placed after the emission filter. The computer used for all experimental studies has the following specifications (Intel Core i7–8700k, 16GB DDR4 2666 MHz RAM, Nvidia Geforce GTX 1070 8GB DDR5 2048 CUDA Cores, Memory Bus 256 Bit). A series of image pre-processing steps, i.e. histogram matching and median filtering, are performed before SIM image reconstruction algorithm, for detailed information about the pre-processing steps please see Section 4 in S1 File.

**Fig 3:**
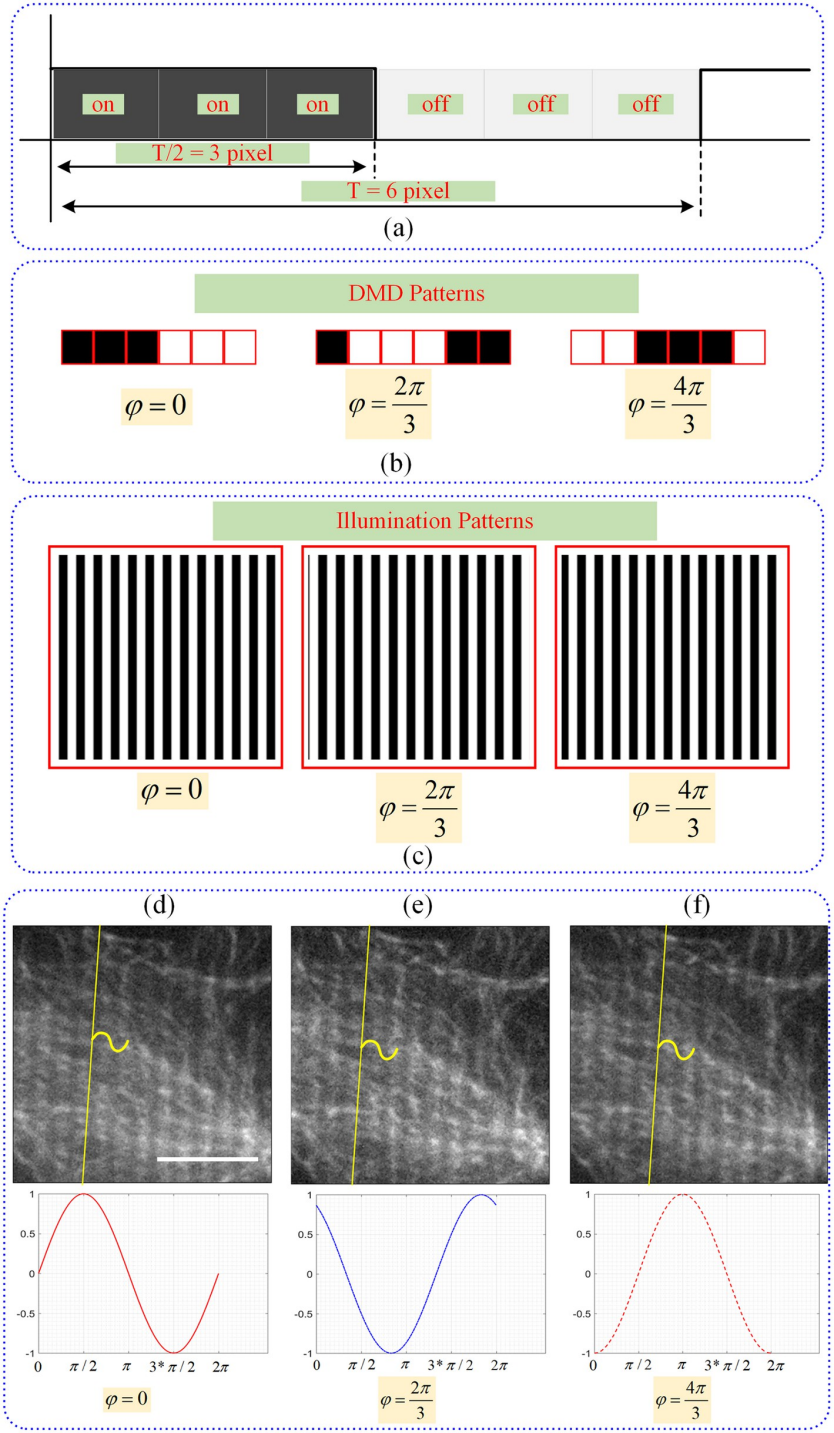
Generating SIM illumination patterns with the DMD chip. (a) Representation of six consecutive DMD mirrors that used for creating illumination patterns. (b) Binary patterns loaded on the DMD in one period of six pixels at three different phases of $\varphi =0,\;\frac{2\pi }{3}\;\frac{4\pi }{3}$, respectively. (c) Corresponding illumination patterns with the same angular orientation and phase values of $\varphi =0,\;\frac{2\pi }{3}\;\frac{4\pi }{3}$, respectively. Cos7 cells were labeled with Alexa-488 (alpha-tubulin, microtubule marker). Microtubule images acquired using three different illumination patterns with the phase values of (d) *φ* = 0, (e) $\varphi =\frac{2\pi }{3}$, (f) $\varphi =\frac{4\pi }{3}$ are shown. The graphs and sketches below the images show the corresponding sinusoidal illumination patterns.

For SIM pattern generation using DLP LightCrafter DLP3000 Evaluation Module, a function called *patternGenerator* (see S5 and S6 Figs in S1 File) has been developed. This function takes phase and period values as parameters and creates illumination patterns in binary image format using these values. The generated patterns are in fact square wave functions since the DMD pixels have only two illumination states. The pattern, when projected onto the sample, will be smoothed out due to the PSF and turn into a sinusoidal pattern. Eq 7 is used to create the illumination patterns and there is a *φ*_*n*_ = 120° phase difference between each illumination pattern. Each of the mirrors on the DMD correspond to a pixel in an illumination pattern image. To create a phase difference of *φ*_*n*_ = 120° between the three illumination patterns in each angular orientation, illumination patterns are created on the DMD chip as a binary image with a minimum period of six pixels (three pixels on, three pixels off). DMD pixels must be at least 6 px/period to create a *φ*_*n*_ = 120° phase difference between each illumination pattern. Fig 3 illustrates how illumination patterns are created by programming DMD mirrors.

As shown in Fig 3(b), all pixels are shifted horizontally by 2 positions to create a *φ*_*n*_ = 120° phase difference between the three illumination patterns. Fig 3(c) shows three illumination patterns with the same angular orientation and a phase difference of *φ*_*n*_ = 120° between them.

## 4 Estimation of the experimental parameters and SIM reconstruction

Phase shift estimation and illumination frequency estimation processes, which are two important steps of the developed image reconstruction algorithm, are explained in this Section. Phase shift estimation is achieved with step following peak finding in the spatial frequency domain. The modulation frequency estimation of illumination patterns is calculated with phase only correlation [24]. In this Section we describe the steps followed for both estimation processes in detail. Accurate determination of these experimental parameters is one of the most crucial steps in SIM reconstruction. These parameters should be accurately determined for each of the 9 illumination patterns prior to SIM reconstruction as ill-determined parameters often lead artefacts in the reconstructed final images [25, 26] (see S1 Fig in S1 File).

### 4.1 Estimation of experimental phase shift

During SIM image acquisition, the sample is sequentially illuminated with periodic illumination patterns with different phase values and orientation angles as specified in Eq 7. For image reconstruction, each spectral component corresponding to unique ***k***_***θ***_ values must then be separated. After each spectral component in Eq 11 is solved using the phase information of the illumination patterns that modulate the raw images, the center points of the images $\tilde{S}\left(k\right)$, $\tilde{S}\left(k-k_{\theta }\right)$ and $\tilde{S}\left(k+k_{\theta }\right)$ are calculated for 0, − ***k***_***θ***_, +***k***_***θ***_ orientations. The phase shift of illumination patterns in a specific region of raw images obtained in a *θ* = 0° orientation and modulated with three separate phase values is schematically shown in Fig 3. The region marked with a yellow line on the images in Fig 3 shows the beginning of the period of the illumination pattern that modulates the image. As seen in the resulting images, a phase difference of *φ* = 120° exists between the illumination patterns on the image. At the bottom of Fig 3(d)–3(f) the positions and states of the DMD mirrors which are used for the generation of the corresponding illumination patterns are indicated.

The phase values cannot be assumed to be equal to those defined for the DMD illumination patterns, and should be experimentally extracted from detector images. Each DMD illumination pattern is originally constructed with a phase shift of *φ* = 0°, 120° and 240°. The actual phase however, depends on the optical pathway and the DMD position relative to the sample and should be determined experimentally. In SIM literature, different methods are reported for phase shift estimation from detector images. K, Wicker *et al*. proposed an iterative method for phase shift estimation with cross-correlation [27]. The method comes together with a relatively high computational cost due to its iterative nature. In addition to iterative phase shift estimation, K. Wicker *et al*. proposed a method that makes phase shift estimation with non-recursive auto-correlation [28], and the method may produce erroneous results when the spatial frequency of the illumination pattern is low. If the resulting raw images have low SNR or high background blur and the presence of noise from imaging devices (e.g. camera readout noise), the performance of image reconstruction will degrade considerably and will cause residual artefact to occur in the final image [28]. In this study, the phase shift calculation was performed by estimating peak positions in the spatial frequency domain. The developed method is known as “phase of peak” in the literature, and has been used in different studies [29]. Here, the performance of this method has been further increased by the approach used in finding the Fourier peaks and reduction of the overall processing time as a result of GPU acceleration.

In order to limit the search area in finding peaks in the Fourier transform of a detector image, a high-pass filter, binary mask filter and a low-pass filter were applied. After this step, peak positions, ***k***_***n-peak***_, were found in the limited search and the corresponding phase values, *φ*_*n*_, were calculated as:

$$\begin{array}{c} \varphi_{n}=tan^{-1}\{\frac{Im[{\tilde{D}}_{n}\left(k_{n-\text{peak}}\right)]}{Re[{\tilde{D}}_{n}\left(k_{n-\text{peak}}\right)]}\}\;. \end{array}$$ (12)

**Fig 4:**
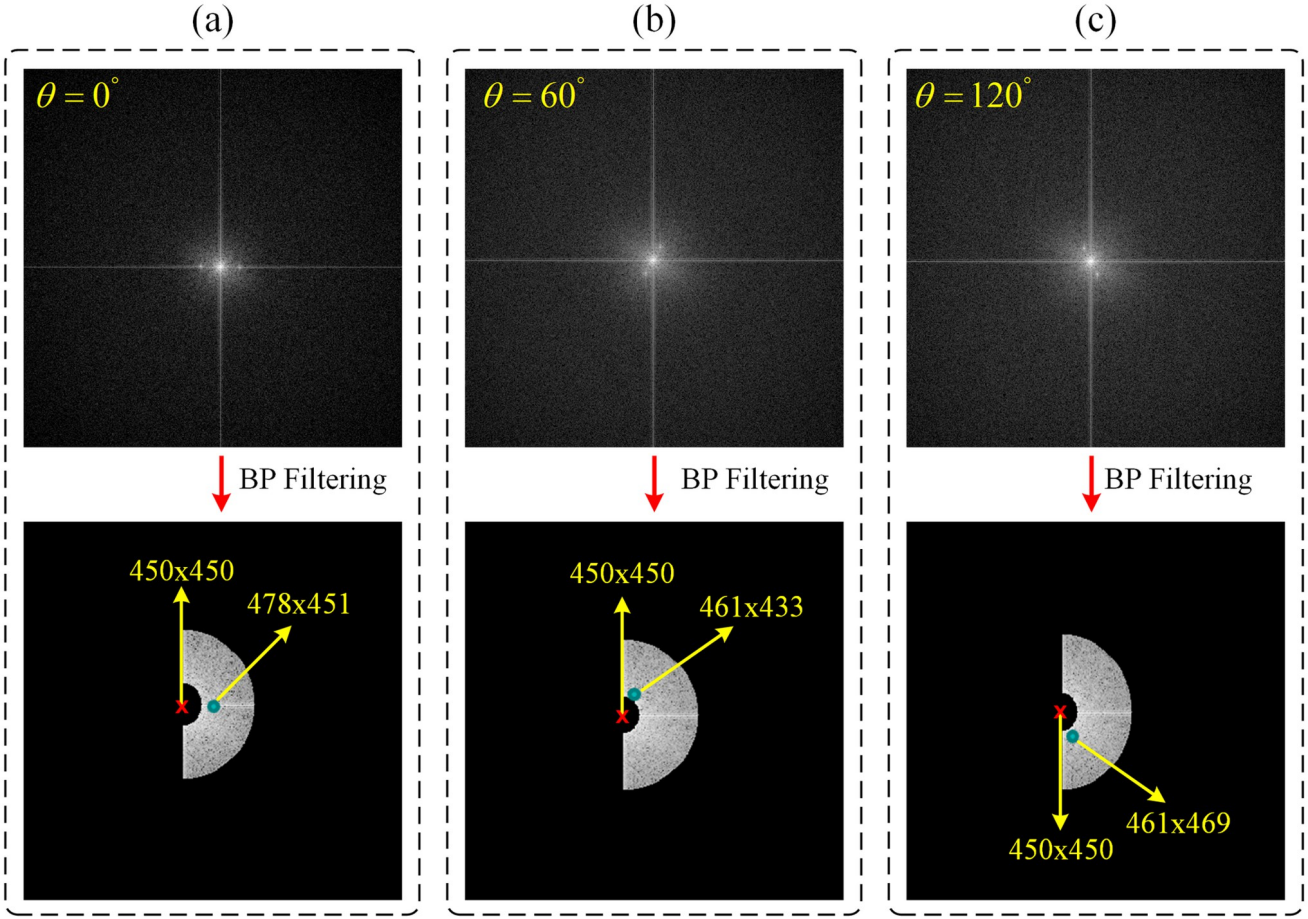
2D spatial frequency spectra of raw images obtained with DMD patterns generated with *φ* = 120° and different *θ* values of 0°, 60°, and 120° are shown in (a), (b), and (c), respectively. Second row shows the same 2D spatial frequency spectra after band-pass filtering. Peak finder algorithm reveals experimental *φ* and *θ* values of (*θ* = −2.04°, *φ* = −21.6°), (*θ* = 57.09°, *φ* = 144.19°), (*θ* = −120.06°, *φ* = 75.3°) for (a), (b), and (c), respectively.

Exemplary two-dimensional (2D) spatial frequency spectra analyzed for extraction of the phase shifts and angular orientations from peak positions are shown in Fig 4.

The fluorescence images that reveal these 2D spatial frequency spectra following Fourier transform are discussed later in Fig 8. The DMD patterns used to obtain Fig 4(a)–4(c) had the same phase shift of *φ* = 120°, and three different angular orientations of *θ* = 0°, 60°, and 120°, respectively. 2D spatial frequency spectra in the second row in Fig 4 show the peak positions, ***k***_***n-peak***_, with respect to the origin after band-pass filtering operation.

The phase shifts can be obtained through Eq 12. The process of finding phase shifts and peak positions is repeated for all images in each angular orientation, thus a total of nine phase values and peak positions are obtained for three different angular orientations. For the specific case shown in Fig 4 our peak finding algorithm reveals experimental (*θ*, *φ*) values of (−2.04°, −21.6°), (57.09°, 144.19°), and (−120.06°, 75.3°) corresponding to those numerically defined in DMD patterns of (0°, 120°), (60°, 120°), and (120°, 120°) in (a), (b), and (c), respectively. The first pixel of the DMD doesn’t correspond to the first pixel of the camera. Hence the phase of the sinusoidal is shifted.

### 4.2 Estimation of experimental spatial frequency vector of an illumination pattern

In our SIM implementation, each illumination pattern was created as a 6 px/period binary image in order to create a *φ* = 120° phase difference between the illumination patterns. During the execution of the SIM image reconstruction algorithm, each frequency component should be shifted to their correct positions after the separation of frequency components with the solution of Eq 11. Using the Fourier shift theorem given below, the separated frequency components obtained from the solution of Eq 11 are shifted by an amount of the spatial frequency vector ***k***_***θ***_ of the illumination pattern [30].

$$\begin{array}{c} F[F^{-1}\{\tilde{S}(k-k_{\theta })\}\times e^{-i2\pi (k_{\theta }\cdot r)}]=\;{\tilde{S}}_{s}\left(k-k_{\theta }\right)\;, \end{array}$$ (13a)

$$\begin{array}{c} F[F^{-1}\{\tilde{S}(k+k_{\theta })\}\times e^{+i2\pi (k_{\theta }\cdot r)}]=\;{\tilde{S}}_{s}\left(k+k_{\theta }\right)\;. \end{array}$$ (13b)

**Fig 5:**
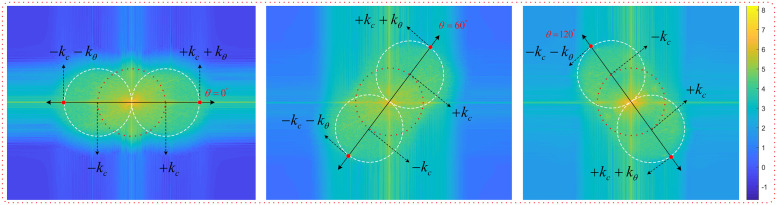
Combined frequency spectrum of images shifted by the *k*_*θ*_ in the angular direction of *θ* = 0°, 60°, 120°, respectively.

In order to calculate the spatial frequency of the illumination pattern, a low-pass filter is applied to each acquired image to suppress the high frequency components outside the *OTF* cutoff frequency, *k*_*c*_ using:

$$\begin{array}{c} {\tilde{D}}_{n-filtered}(k)=\;\frac{{\left(OTF(k)\right)}^{*}\cdot {\tilde{D}}_{n}\left(k\right)}{|OTF(k)|+\sigma }\;, \end{array}$$ (14)

where *σ* is a small positive constant that prevents division by zero error. After the suppression of high frequency noise, the illumination pattern spatial frequency vector is calculated using Eq 11, which separates modulated images with three different phase values. Eq 13 is applied to the separated spectral components in each angular direction (except the DC component), so that the center points of all spectral components are shifted to the origin. The extended frequency spectra of the nine images with *θ* = 0°, 60°, 120° orientations are calculated using Eq 13, and the center points of the spectral components separated in each angular direction are shifted to the origin by an amount of ***k***_***θ***_. This process is illustrated in Fig 5.

### 4.3 SIM reconstruction

After experimental phase shift and spatial illumination frequency values are obtained, nine images are combined to form a single high resolution image. In the final step of the SIM image reconstruction, the generalized Wiener filter was used to generate the high-resolution image. The Wiener filter is a statistical filter model that aims to minimize the mean square error by removing the added noise in general image reconstruction [31]. Equations below are used to combine nine images using the Wiener filter:

$$\begin{array}{c} \begin{array}{c} R_{1}(k)=\;{\tilde{S}}_{1}(k)\;,\\ R_{2}(k)={\;\tilde{S}}_{2}(k-k_{\theta })\;,\\ R_{3}(k)={\;\tilde{S}}_{3}(k+k_{\theta })\;,\\ S_{SIM}\left(k\right)=\;\frac{\sum_{n=1}^{N}{OTF}_{n}{\left(k\right)}^{*}R_{n}\left(k\right)}{\sum_{n=1}^{N}{\left|{OTF}_{n}\left(k\right)\right|}^{2}+w}\;, \end{array} \end{array}$$ (15)

where, *S*_*SIM*_(***k***) represents the Fourier transform of the final reconstructed high resolution image, *OTF*_*n*_(***k***) represents shifted *OTF* corresponding to the *n*^*th*^ image, “*” indicates the complex conjugate operation, and *w* is the Wiener filter constant. The high resolution image in real space *S*_*s*_(***r***) is then obtained by inverse Fourier transform of *S*_*SIM*_(***k***) as:

$$\begin{array}{c} S_{s}(r)=\;F^{-1}[S_{SIM}(k)]\;. \end{array}$$ (16)

## 5 GPU acceleration

Graphics Processing Unit (GPU) provides computation of high resolution graphics in computer systems. Modern high power GPUs have thousands of cores within a single hardware unit, allowing for thousands of hardware threads to be run simultaneously [32, 33]. The high computational power of GPUs and their suitability for parallel processing of data led to their wide adoption in microscopy image processing applications. It has been demonstrated that GPUs provide the expected performance improvement in imaging systems where real-time image reconstruction is required [12, 34–37].

**Fig 6:**
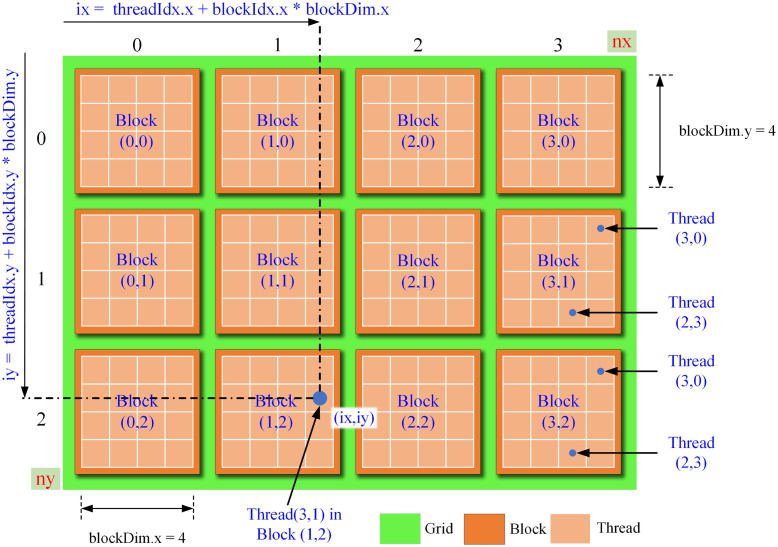
CUDA threading and block organisation of two-dimensional matrix.

Element-wise matrix operations on GPU cores are executed simultaneously with CUDA kernel functions using the developed parallel SIM reconstruction algorithm. For each element-wise matrix operation, a CUDA kernel function has been developed. Each thread in the corresponding CUDA kernel function is configured to access a specific item in the matrix in order to perform element-wise matrix operations with many threads in parallel. As an example, in the MATLAB, the operation *c* = *a* + *b* (where, a, b and c are all matrices of *M* × *N* size) is a matrix addition and the elements of the matrices are added to each other and then written to the corresponding index in the result matrix. A kernel function was designed with SIMD (Single Instruction, Multiple Data) approach to perform this calculation made in MATLAB with the Parallel CUDA kernel function. In multidimensional data, initial block and thread sizes must be determined in accordance with the data type in the CUDA kernel function. Fig 6 shows the organisation of CUDA threads and block sizes designed for a two-dimensional matrix.

In Fig 6, the matrix consists of a total of 12 blocks and each block consists of a total of 16 threads. The thread in each block has a unique block number. Fig 6 shows the calculation of a matrix element’s global index value for Block (1,2). In this structure, there are a total of 192 threads that operate simultaneously with the Matrix items. Since these threads can work simultaneously, in the process of adding two-dimensional matrices to each other, the sum of each element is carried out in parallel by a different thread. In this study, several operations on 2D image matrices are performed using CUDA kernel functions. To begin with, the parameters for each kernel’s launch process should be determined. Each kernel data is a 2D image matrix. For CUDA kernel function to be executed it is necessary to define how many blocks the kernel function will have and how many threads will be in total in each block. Size of grids and blocks is calculated as follows. Suppose we have two variables, *dimx* and *dimy*, at x and y to determine the number of threads in each block. Grid parameter defines how many blocks the data will consist of. In addition, when determining *ThreadBlockSize* and *GridSize* values, the image matrix dimensions to be calculated should be considered. For example, let *szX* and *szY* be the dimensions of the image matrix, and *dimx* and *dimy* are the frame size of each thread block in this matrix (it is recommended to set *ThreadBlockSize* as 16 or 32), the *GridSize* covering all image matrix elements is as follows must be calculated. *grid* = [*ceil*(((*szX* + *dimx* − 1)/*dimx*))*ceil*(((*szY* + *dimy* − 1)/*dimy*))];. *dimx* and *dimy* are set to 32 and show the total number of threads in a tile. More detailed explanation on kernel functions and kernel launches is provided in S1 File.

In this study, thousands of CUDA hardware threads were used to calculate raw image data in the parallel image reconstruction algorithm developed for SIM, and a general purpose toolbox was created in MATLAB for the developed parallel SIM image reconstruction algorithm. This toolbox enables the user to perform all calculations on the image data in parallel via the GPU cores. An explanation of the developed parallel CUDA kernel functions and the definitions of these functions can be found in the S1 Table in S1 File. All codes are also available in https://github.com/msaaydin/SIM1 repo.

**Fig 7:**
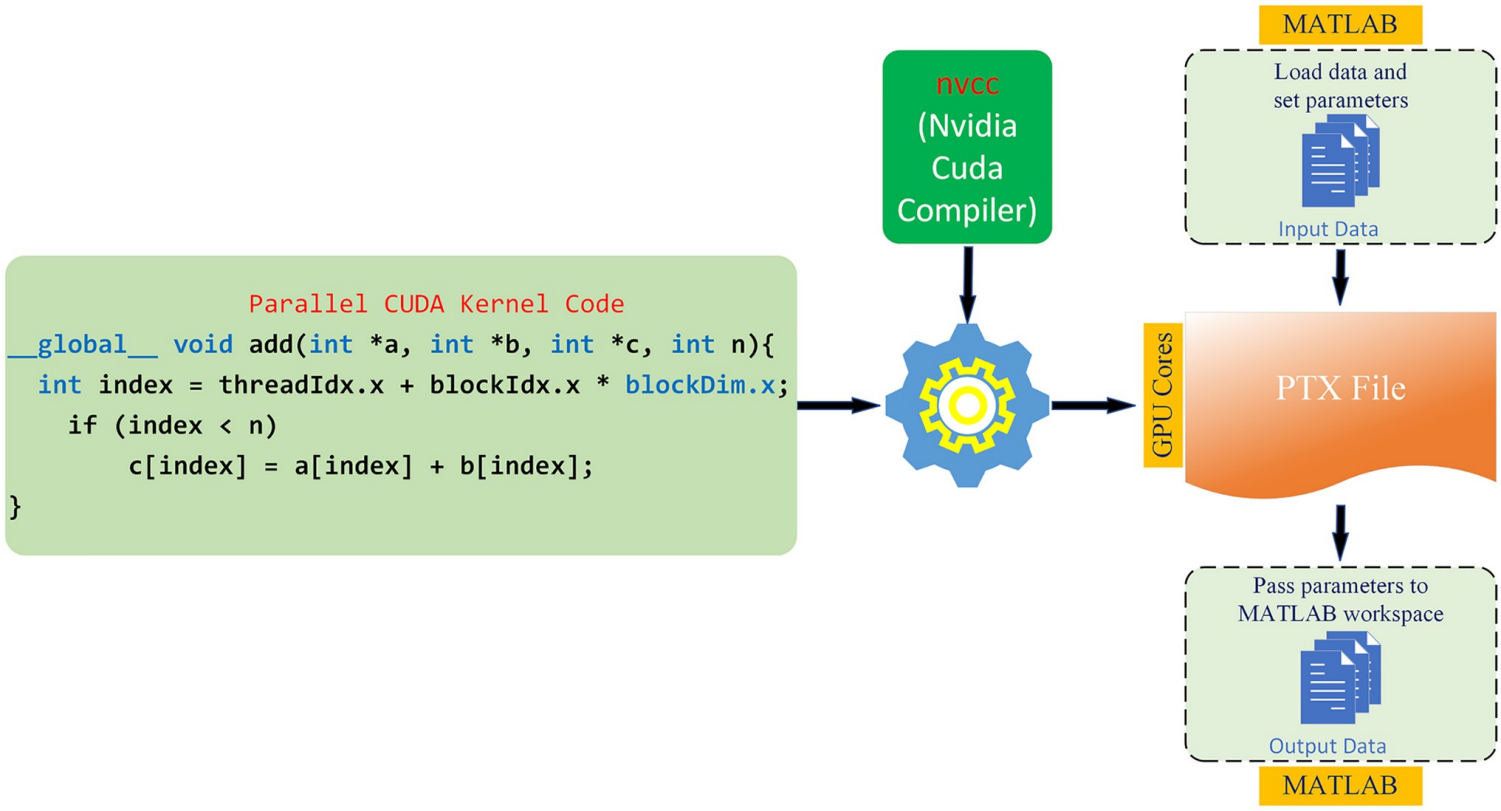
The diagram of parameter passing and communication architecture between MATLAB-CUDA.

The schematic diagram of the parameters passing between the developed CUDA parallel kernel functions and MATLAB variables is shown in Fig 7 together with the flowchart.

First, a CUDA kernel function is developed to perform calculations with the GPU, and then a MATLAB *mex* function is designed to pass parameters between MATLAB and the CUDA kernel function. The image data in MATLAB is passed as a parameter using the *mex* function. After the parameters are sent, space is allocated for this data in the GPU memory and the data is copied from the CPU memory to the GPU memory. The CUDA kernel function designed for GPU computation is compiled with *nvcc* and the CUDA kernel function is executed in parallel with the GPU cores. The results of the data run with the GPU are copied back to the host memory (CPU memory) and the previously allocated memory areas in the GPU memory are freed. The list of functions optimized with CUDA is provided in Section 2 of the S1 File.

## 6 Results

**Fig 8:**
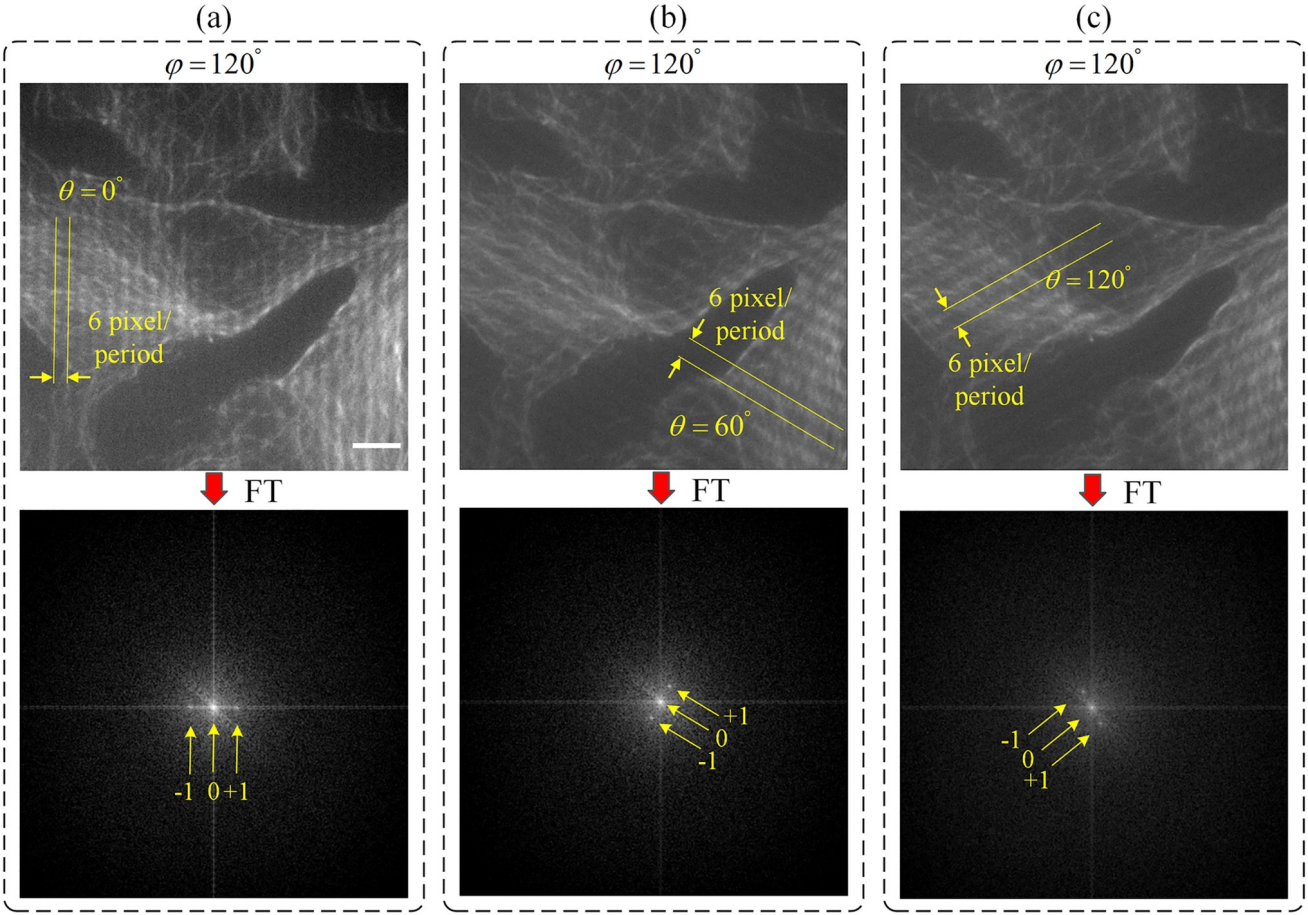
Cos7 cells were labelled with anti-tubulin and secondary Alexa-488. Raw fluorescence images of microtubules modulated with sinusoidal illumination patterns with the same phase angle of *φ* = 120°, and three different orientation angles of *θ* = 0°, 60°, are shown in (a), (b), and (c), respectively. Bottom row shows the frequency spectra of the corresponding raw images at the top row. Scale bar indicates 5 *μ*m.

Raw images are used for obtaining single super-resolution images with the developed SIM reconstruction algorithm using both GPU computation and CPU computation. Before SIM reconstruction, image pre-processing consisting of histogram matching and median filtering steps is performed on raw images (see S1 File for more details). Developed SIM reconstruction algorithm was initially tested on an artificial image, and the algorithm’s accuracy was validated. Section 6 in the S1 File contains the results of the artificial image test. Fig 8 shows exemplary raw images illuminated by sinusoidal illumination patterns at 6 px/period spatial frequency with the same phase shift value of *φ* = 120°, and three different orientations corresponding to *θ* values of 0°, 60°, and 120°).

**Fig 9:**
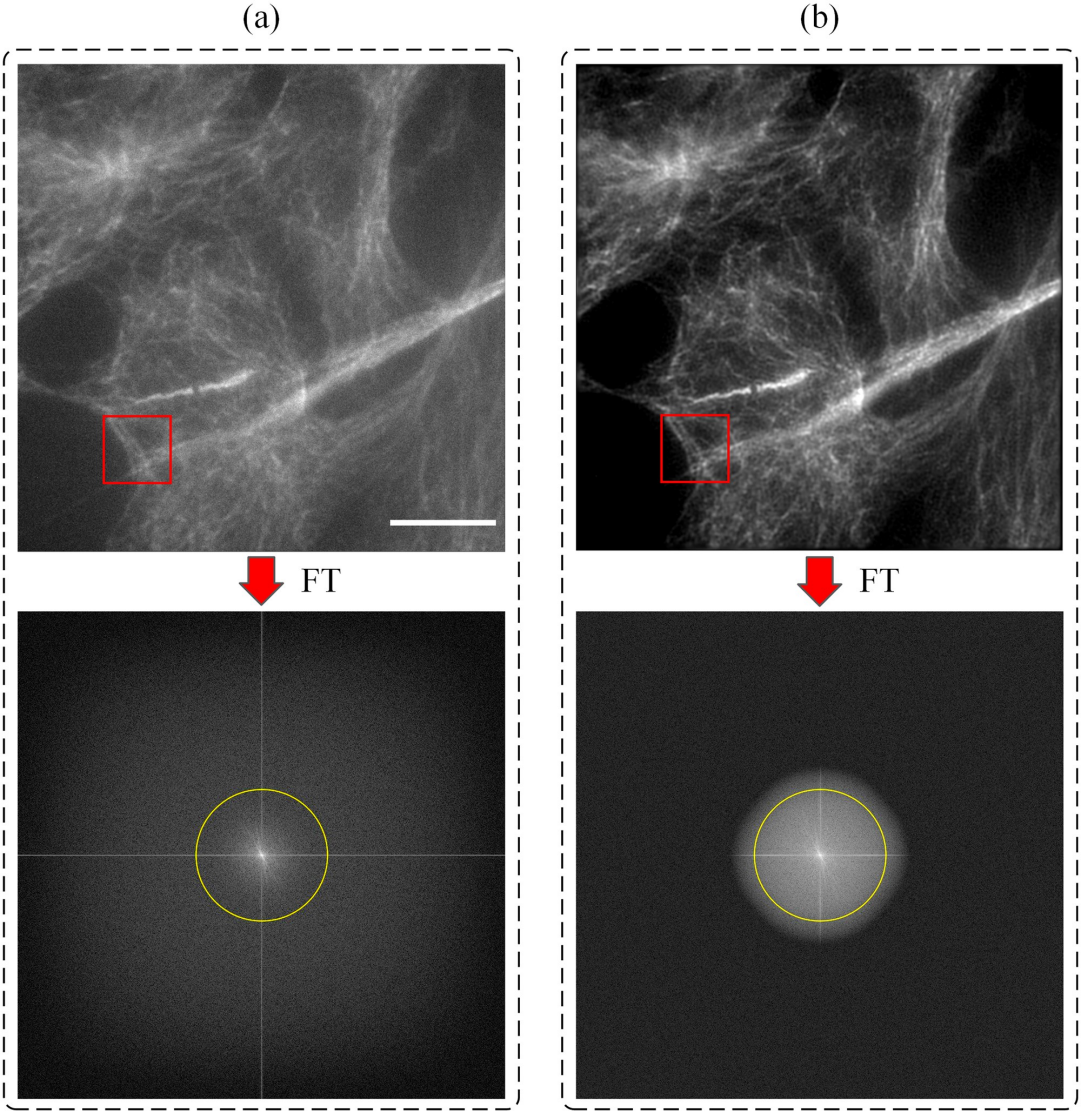
Cos7 cells were labelled for tubulin (Alexa Fluor 488). Microtubule images obtained with (a) wide-field illumination and (b) SIM reconstruction are shown together with their Fourier transforms. Scale bar indicates 10 *μ*m. Yellow circles indicates the limits of assumed OTF.

2D spatial frequency spectra of these raw images are also shown in Fig 9. Peaks corresponding to illumination pattern modulations are clearly visible in 2D spatial frequency spectra.

Fig 9 shows an exemplary pair of images obtained with wide-field illumination and SIM reconstruction, together with the corresponding 2D spatial frequency spectra.

The image with wide-field illumination image is recorded by keeping all DMD mirrors in their *on* state. For the image obtained using wide-field illumination (Fig 9(a)), it is seen that the 2D spatial frequency spectrum is dominated by relatively low spatial frequency components. On the other hand, Fig 9(b) shows that with the usage of SIM, the observable bandwidth in the 2D spatial frequency spectrum has increased considerably, and as such, more information from the high frequency components is included in the image.

**Fig 10:**
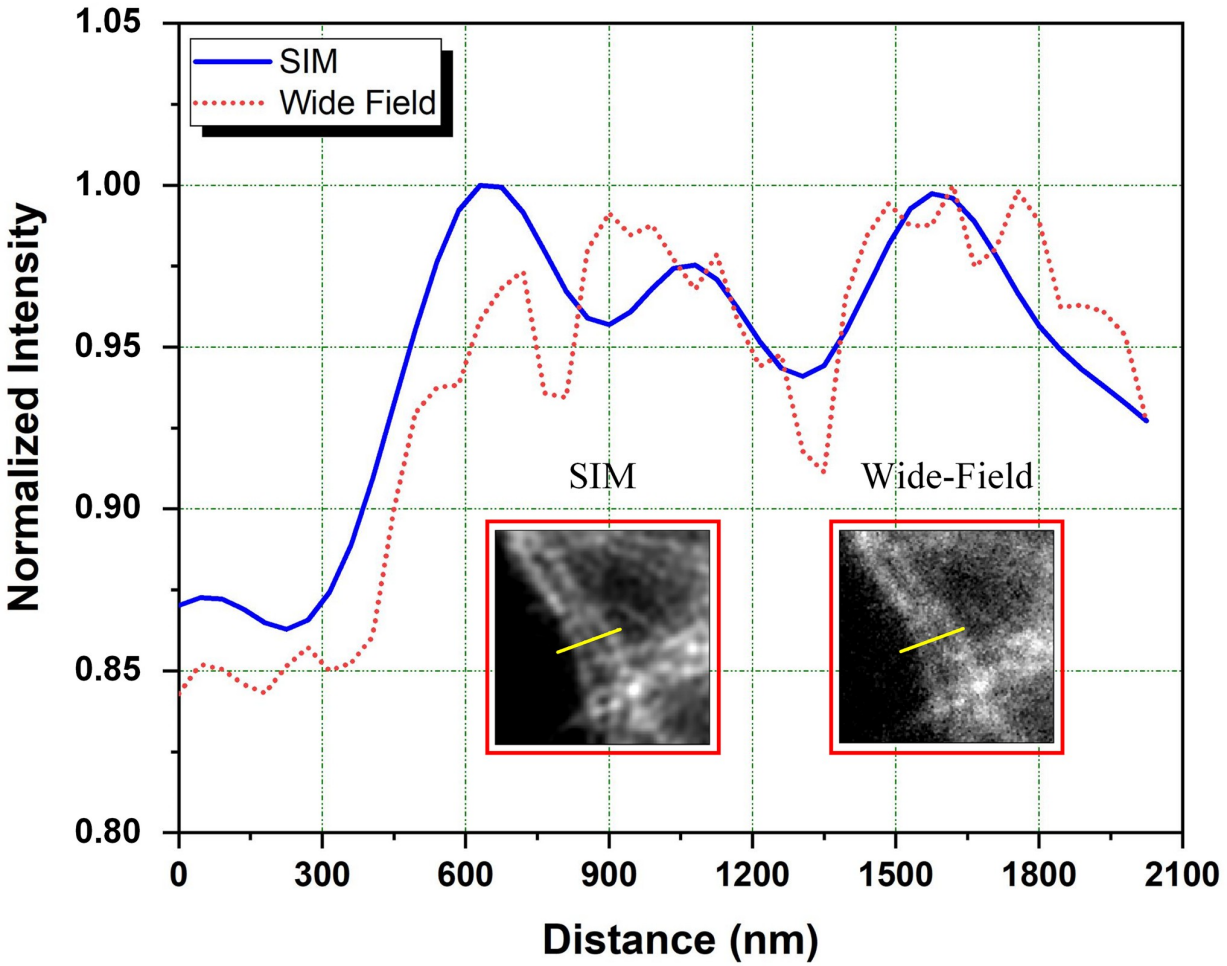
Line profiles of intensity values in areas indicated with red boxes in Fig 9. Insets show the zoomed wide-field and SIM images of microtubules. Line profiles were plotted along yellow lines presented in the figures.

Line profiles of intensity changes along the yellow lines inside regions indicated with the red boxes in Fig 9(a) and 9(b) are shown in Fig 10. Line profile obtained from the SIM image reveals clearer details with a higher feature contrast as compared to the line profile obtained from the image obtained using wide-field illumination where the details are lost and the image becomes blurred (see Fig 10).

**Fig 11:**
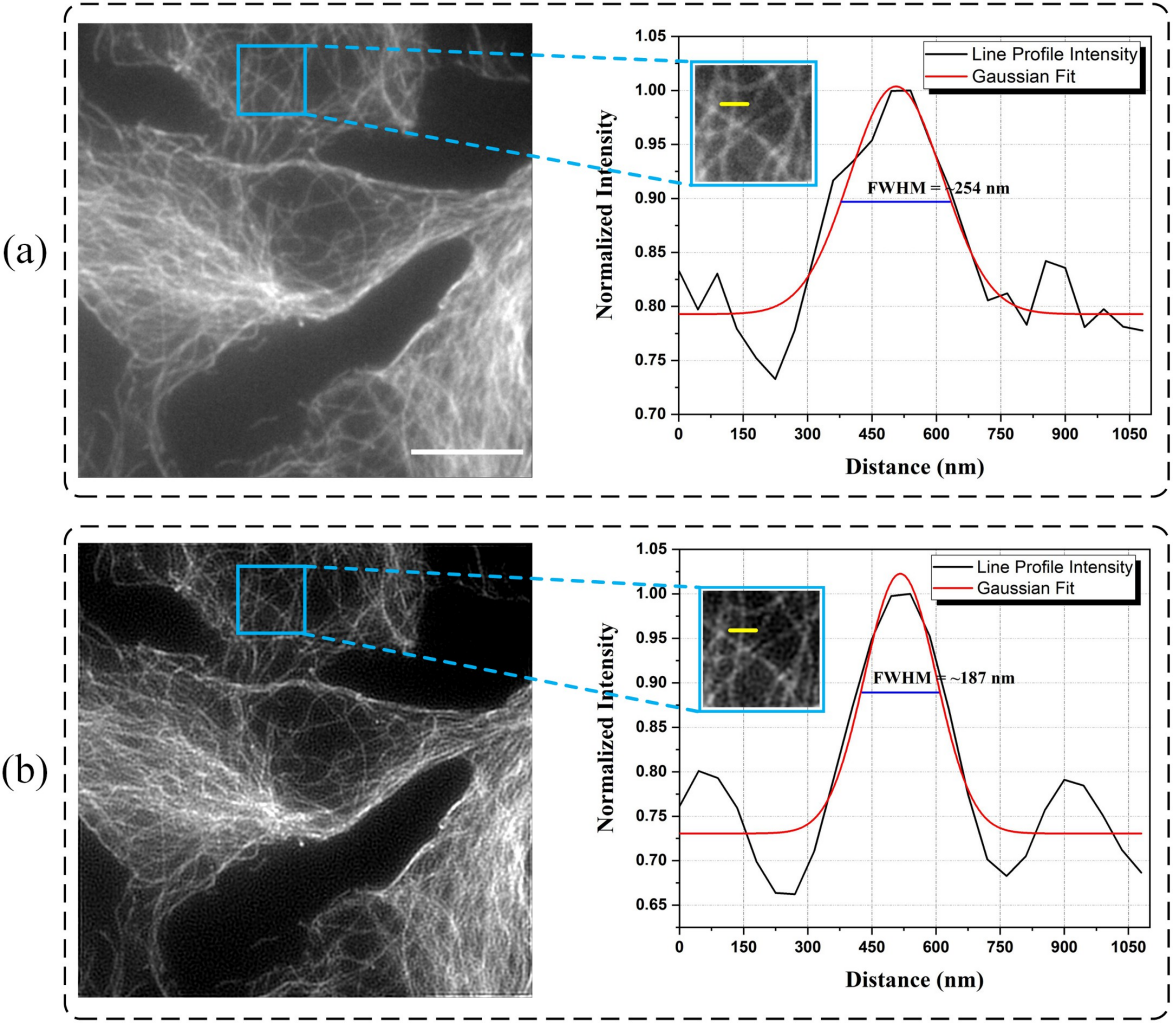
Cos7 cells were labelled for tubulin (Alexa Fluor 488). Microtubule images obtained with (a) wide-field illumination and (b) SIM reconstruction together with line profiles in selected regions. Line profiles were plotted along yellow lines presented in the insets. Scale bar indicates 10 *μ*m.

Another pair of images obtained by wide-field illumination and SIM reconstruction is shown in Fig 11.

As a result, the DMD plane’s spatial frequency is 6 px/cycle. The spatial frequency on the sample plane was measured at 33 px/cycle (The pixel size in the simulated raw image corresponds to 45 nm in sample space, therefore, the resolution in the sample plane is calculated as 1485 nm/cycle). This is the maximum spatial frequency that can be achieved. Line profiles obtained from the regions indicated by blue boxes show minimum feature sizes of microtubules observed in those cases. Line profile obtained from the wide-field illuminated image reveals a FWHM of ∼254 nm while a FWHM of ∼187 nm is obtained from the SIM reconstructed image.

**Fig 12:**
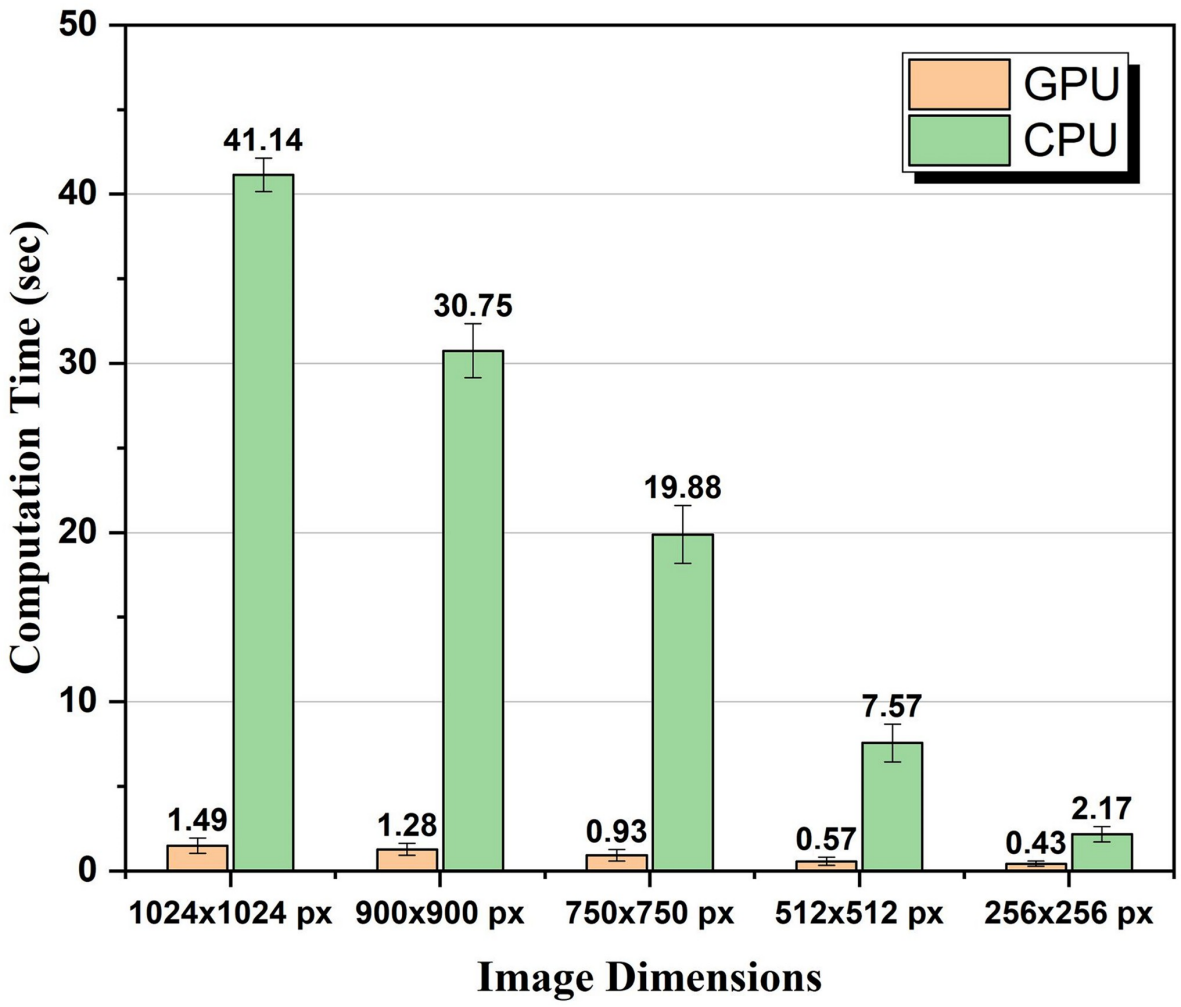
Total GPU and CPU computation times of SIM reconstruction algorithms executed using images with different sizes.

In order to quantify the improvement in computation time caused by GPU acceleration, SIM reconstruction was performed using GPU computation and CPU computation for different images with different dimensions. Total computation times elapsed for different cases are shown in Fig 12.

Fig 12 shows the total execution times of all steps in the SIM reconstruction algorithm including reading images, data transfers between CPU and GPU, performing all calculations, and plotting of the results. For CPU and GPU algorithms execution times given in Fig 12, all algorithms were run 10 times on both CPU and GPU, and the average of these times determines the execution time. As a result, 28, 25, 22, 14, and 6 times faster computation is observed for GPU computation in contrast to mono-thread CPU computation for image dimensions of 1024 × 1024 px, 900 × 900 px, 750 × 750 px, 512 × 512 px, and 256 × 256 px, respectively. Such a performance improvement can not be achieved using a multi-core CPU parallel threads [38, 39]. We also tested our mono-thread CPU algorithm with multi-thread OpenMP CPU using 12 threads and 6 CPU cores [40]. As a result, 20, 18, 14, 12, and 5 times faster computation is observed for GPU computation in contrast to multi-thread OpenMP CPU computation for image dimensions of 1024 × 1024 px, 900 × 900 px, 750 × 750 px, 512 × 512 px, and 256 × 256 px, respectively. Computation time improvements by less than a factor of 1.6 for mono-thread CPU algorithm were observed for all images dimensions. These improvements are much less significant than improvements by up to 28 times observed as a result of GPU acceleration in our work. For the case of 512 × 512 px, we also tested the execution times of the algorithm with OpenSIM [26] and built-in gpuArray enabled functions in MATLAB. Total execution times for SIM image reconstruction of 512 × 512 px images were measured 68 sec and 3.82 sec using OpenSIM and gpuArray, respectively. These execution times are much larger than the total execution time of 0.628 sec reached with our CUDA based implementation for 512 × 512 px images.

## 7 Conclusion

SIM employs sinusoidal modulated fluorescence microscopy images with shifted spatial frequency components. By means of a reconstruction algorithm, it is possible to obtain a reconstructed fluorescence image with spatial frequency components reaching beyond the critical frequency set by the diffraction barrier of the overall imaging system. This approach is favourable to other super-resolution fluorescence microscopy methods because it enables relatively fast image reconstruction, and requires relatively low cost modifications in the setup of a conventional wide-field fluorescence microscope. In this work, we realized such a compact experimental setup that employs an LED illuminated DMD projector for intensity modulation of the illumination patterns. The use of an LED illuminated projector provides a low cost approach avoiding the need for expensive components including laser light sources and SLMs while providing sufficient performance on pattern resolution and refresh rate. A SIM reconstruction algorithm was designed that includes pre-processing with histogram matching and median filtering, extraction of experimental parameters including the phase shift and spatial illumination frequency, and use of the generalized Wiener filter for obtaining the super-resolution image. Furthermore, we also presented a tool consisting of GPU-based parallel CUDA kernel functions that enables parallel executions of SIM reconstruction in MATLAB for general purpose users. The presented tool is compatible with all NVIDIA GPUs. Our experiments performed with Cos7 cells labelled for tubulin with Alexa FLuor 488 revealed significant improvements in contrast and minimum feature size observed in SIM reconstructed images in contrast to images collected by wide-field illumination. For different image dimensions we evaluated the computation times obtained with our tool based on GPU acceleration, standard approach using a mono-thread CPU and multi-thread OpenMP CPU. We observed up to ∼28 and ∼20 times speed up in SIM reconstruction when mono-thread CPU vs GPU and multi-thread OpenMP CPU vs GPU are used respectively for image dimensions of 1024 × 1024 px. The demonstrated algorithmic and optical design of a low-cost, portable, super-resolution imaging system for SIM can be extended with the use of a higher resolution DMD, and a more compact mechanical design. Future improvements to our approach may include extension to multiple wavelength acquisition, 3D optical sectioning, high-speed subcellular live imaging, and incorporation of machine learning approaches for SIM reconstruction [41].

### 8 Materials

COS-7 (African green monkey kidney cells, CRL1651; ATCC) were cultured with Dulbecco’s modified Eagle’s Medium DMEM/F12 50/50 medium (Pan Biotech, Vienna, AUT) supplemented with 10% Fetal Bovine Serum (FBS, Life Technologies, Carlsbad, Ca, USA) and 1% penicillin-streptomycin (Gibco, Thermo Fisher Scientific) with 5% CO2 in 37° *C*. Cell line was tested for mycoplasma by MycoAlert Mycoplasma Detection Kit (Lonza, Basel, CH). The S1 File contains detailed information of the material (samples) used in this study.

## Supporting information

S1 File Supplementary material to the manuscript. (PDF)
