# Supplementary material for: An LED-Based structured illumination microscope using a digital micromirror device and GPU accelerated image reconstruction
Source: PLoS One. 2022 Sep 9;17(9):e0273990. doi: 10.1371/journal.pone.0273990 (PMC9462783; doi:10.1371/journal.pone.0273990)
Supplement: S1 File — (PDF) [file pone.0273990.s001.pdf]

Supplementary Information

for

An LED-Based Structured Illumination Microscope Using a Digital Micromirror Device and GPU Accelerated Image Reconstruction

Musa Aydın [1*], Yiğit Uysallı [2], Ekin Özgönül [2], Berna Morova [2,3], Fatmanur Tiryaki [4], Elif Nur Firat-Karalar [4,5], Buket Doğan [6], Alper Kiraz [2,3,7*]

[1] *Department of Computer Engineering, Fatih Sultan Mehmet Vakıf University, Istanbul, Turkey*

[2] *Department of Physics, Koç University, Istanbul, Turkey*

[3] *KUTTAM, Koç University Research Center for Translational Medicine, 34450 Istanbul, Turkey*

[4] *Department of Molecular Biology and Genetics, Koç University, 34450 Istanbul, Turkey*

[5] *School of Medicine, Koç University, 34450, Istanbul, Turkey*

[6] *Department of Computer Engineering, Marmara University, Istanbul, Turkey*

[7] *Department of Electrical and Electronics Engineering, Koç University, Istanbul, Turkey*

* Corresponding authors: maydin@fsm.edu.tr, akiraz@ku.edu.tr

1. SIM image reconstruction analysis

In the image reconstruction process, the spatial frequency of the illumination pattern is used to shift the separated frequency components in the frequency domain to correct positions obtained by solving Equation 11 in the main manuscript. Shifting is performed by the magnitude and direction of the calculated spatial frequency vectors $\boldsymbol{k}_{\theta_1}, \boldsymbol{k}_{\theta_2}, \boldsymbol{k}_{\theta_3}$ in each angular orientation of the illumination pattern. When the illumination pattern's spatial frequency is computed improperly, the spectral components overlap incorrectly.

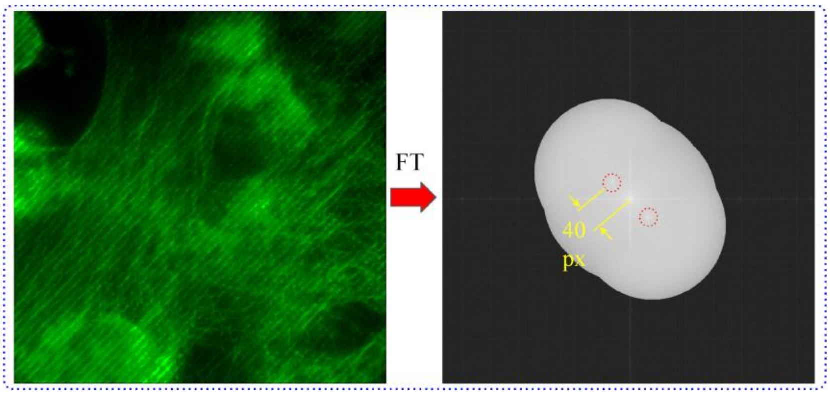

Figure S1. The noisy image and the 2D spatial frequency spectrum of the image obtained as a result of incorrect calculation of the spatial frequency vector of the illumination pattern.

Figure S1 shows the results of an incorrect spectral shifting. For the image given in Figure 1, SIM reconstruction used a spatial frequency vector that was incorrectly calculated. As a result the spectral components in 60 degrees orientation could not be shifted to their correct positions in the high-resolution image, the frequency components did not overlap and a periodic noise occurred in the final image. You can access all of the developed parallel CUDA functions from this **github** (https://github.com/msaaydin/SIM1) repo.

2. GPU based SIM image reconstruction algorithm

Table S1 contains the list of CUDA kernel functions created for matrix calculations performed for SIM reconstruction. The definition of the exemplary CUDA kernel function that performs **c = a .* b** from the functions in Table S1 is given in Figure S2. In the "**cudapointProductMatrixGPU**" function mentioned in Figure S2, the elements of MatA and MatB matrices are multiplied separately, and the result is written to the corresponding index value of the MatC matrix. The function takes five parameters. First and second parameters (const double *MatA, const double *MatB) are input matrices while the third parameter (double *MatC) is the result matrix. The fourth and fifth parameters specify the dimensions of the matrices. The keyword const at the beginning of the matrix sent as a parameter indicates that the specific matrix constitutes an input argument.

Table S1. List of CUDA kernel functions developed for SIM reconstruction.

Kernel Function Name	Operation	Definition
**List of kernel functions for matrices with real numbers**		
cudaSum	c = a + b	a and b are real matrices, it calculates the sum of the two matrices.
cudaSub	c = a - b	a and b are real matrices, subtract the two matrices from each other.
cudapProductMatrixGPU	c = a .* b	a and b are real matrices multiplied by each other element by element.
cudaMatrixSumReduction	s = sum(sum(a))	a is a real matrix, calculates the sum of all the elements of this matrix with dimension reduction in $log_2 n$ steps.
cudaMatrixMult	c = a * b	a and b are real matrices, calculates the multiplication of the two matrices
cudaInnerDiv	c = a ./ b	a and b are real matrices, dividing the two matrices element by element
cudaElementSquere	c = a .^ 2	a is a real matrix, it calculates the square of all the elements of this matrix.
cudaElementSqrByvalue	c = a .^ m	a is a real matrix, calculates the m nth exponent of all the elements of this matrix.
cudaElementProd	c = a .* m	a is a real matrix, all the elements of this matrix are multiplied by a constant m.
cudaMatrixBinariseG	c = a > const	a is a real matrix, it compares all the elements of this matrix with the const constant, and assigns the value to the new matrix as “1” if it is large, otherwise as “0”.
cudaMatrixBinariseL	c = a < const	a is a real matrix, it compares all the elements of this matrix with the const constant, and assigns the value to the new matrix as “0” if it is large, otherwise as “1”.
**List of kernel functions for matrices with complex numbers**		
cudaComplexAdd	c = a + b	a and b are complex matrices, calculates the sum of the two matrices
cudaComplexDiv	c = a ./ b	a and b are complex matrices, dividing the two matrices element by element
cudaComplexSub	c = a - b	a and b are complex matrices, subtract the two matrices from each other
cudaComplexMul	c = a .* b	a and b are complex matrices, multiplied by each other element by element.
cudaComplexConj	c = conj(a)	a is a complex matrix, calculates the complex conjugate of this matrix

cudaComplexAbs	c = abs(a)	a is a complex matrix, Calculates the complex magnitude of all the elements of this matrix
cudaComplexMakeBinaryMat	c = a > const	a is a complex matrix it compares all the elements of this matrix with the const constant, and assigns the value to the new matrix as “0” if it is large, otherwise as “1”.

A list of CUDA kernel functions developed to be used in the calculations for the SIM reconstruction algorithm is given in Table S1.These CUDA kernel functions given in Table S1 are used in different parts of the reconstruction algorithm as needed. To give an example of these;

- The equations S2 and S3 (in the supplementary file), which are used for phase shift estimation and illumination frequency estimation calculation, are calculated on the GPU with the CUDA core functions.
- Dot product of the original image with the ring (notch) filter for phase shift estimation (im = im.*filter;)
- The max() CUDA kernel function, which was developed to find the max value of the peaks in the Fourier spectrum, was used in the phase shift estimation and illumination frequency estimation stages.
- To compute an image's magnitude spectrum (abs(a) a is a complex matrix, Calculates the complex magnitude of all the elements of this matrix), For example, this calculation is done in Equation S3 in the supplementary file, firstly the wide area image is multiplied by a constant (c = a .* m, a is a real matrix, all the elements of this matrix are multiplied by a constant m). Using the CUDA kernel function, then subtracting the resulting matrices from each other (c = a - b, see Supplementary Information Table S1)

All arguments without the const keyword at the beginning are defined as arguments to be returned from the function.

```
// c  = a.*b; a and b are 2D matrices
__global__ void pointProductMatrixGPU(const double *MatA, const double *MatB, double *MatC, const int nx, const int ny)
{
    unsigned int ix = threadIdx.x + blockIdx.x * blockDim.x;
    unsigned int iy = threadIdx.y + blockIdx.y * blockDim.y;
    unsigned int idx = iy * nx + ix;

    if (ix < nx && iy < ny)
    {
        MatC[idx] = MatA[idx] * MatB[idx];
    }
}
```

Figure S2. CUDA kernel function that simultaneously multiplies the elements of two 2D matrices.

A **ptx** file is needed to call a CUDA kernel function with MATLAB. The source file with the **"cu"** extension, which contains a CUDA kernel function, is compiled with the CUDA C compiler (Nvidia CUDA Compiler-nvcc) and a **ptx** file is created. Figure S3 shows how to pass a data from MATLAB to the **ptx** file as a parameter. According to the given flowchart, firstly the CUDA kernel function source file is compiled using **nvcc** from the command line and the .**ptx** file is created.

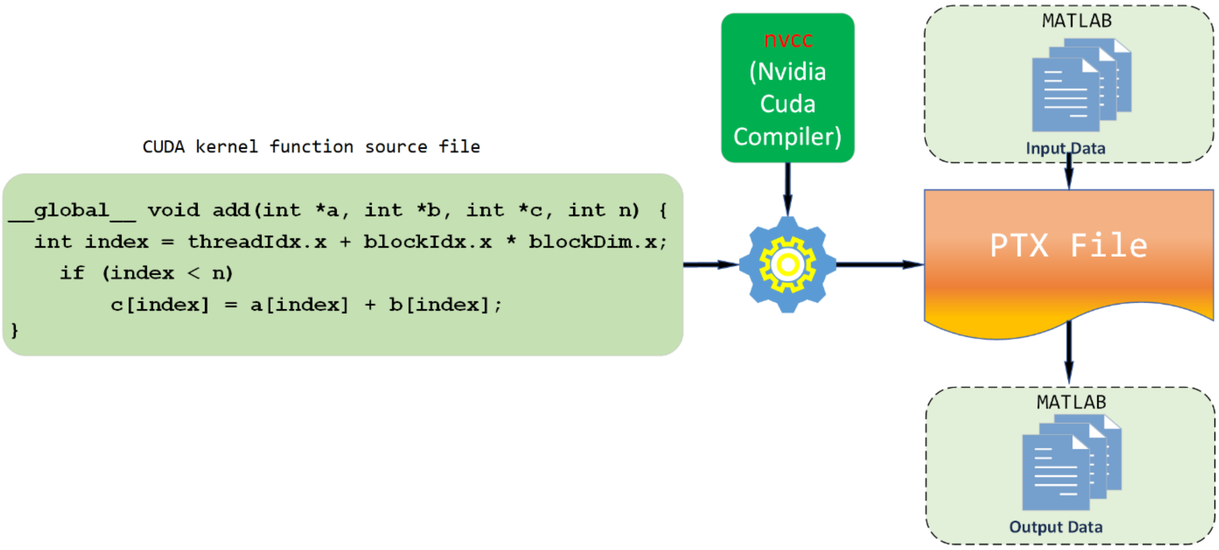

Figure S3. Parameter passing using ptx file to CUDA kernel function with MATLAB.

Using the created **ptx** file, the data in the CPU memory in MATLAB is called in accordance with the parameter list of the kernel function in the source file. In the invocation step, the parameters defined in MATLAB are copied to the GPU memory for processing in the **ptx** file and operated by the GPU in accordance with the rules in the kernel function defined in the GPU. Finally, the result is copied back from the GPU memory to the host side, that is to the CPU memory, to MATLAB, and the calculation is completed. With the following command line, the source file "matOperation.cu" is compiled with the CUDA C compiler and a **ptx** file is created;

```
nvcc -ptx matOperation.cu --gpu-architecture=compute_61 --gpu-code=sm_61
```

In this study, an Nvidia Geforce GTX 1070 graphics card was used for GPU accelerated SIM reconstruction. Descriptions of the parameters in the command line are as follows;

- --gpu-architecture = Compute Capability version
- --gpu-code = Streaming Multiprocessors Capability version,

After the **ptx** file is created, the MATLAB kernel function object is created with the created **ptx** file from the MATLAB command line. By using this kernel function object, the parameters pass to the **ptx** file and the relationship between CUDA and MATLAB is established. After setting the initial values, block size and number of threads in each block of a ptx file compiled with the CUDA C compiler, the kernel function is executed by sending the necessary parameters to the relevant CUDA kernel function. For this process, the kernel function object must be defined in the MATLAB command line as follows.

```
kernelMul = parallel.gpu.CUDAKernel('matOperation.ptx','matOperation.cu',
cudapointProductMatrixGPU');
kernelMul.ThreadBlockSize  = [dimx dimy];
kernelMul.GridSize         = grid;
```

For CUDA kernel function to be executed and parameterized with **kernelMul** kernel function object; it is necessary to define how many blocks the kernel function will have and how many threads will be in total in each block. In the command set shown above, the dimx and dimy parameters define the numbers of threads in the block, and the grid parameter defines how many blocks the data will consist of. In addition, when determining ThreadBlockSize and GridSize values, the image matrix dimensions to be calculated should be considered. For example, if the MATLAB command line given below is considered, let szX and szY be the dimensions of the image matrix, and dimx and dimy are the frame size of each thread block in this matrix (it is recommended to set ThreadBlockSize as 16 or 32), the GridSize covering all image matrix elements is as follows must be calculated.

```
dimx = 32; % tile size X threads per block
dimy = 32;
grid = [ceil(((szX+dimx-1)/dimx)) ceil(((szY+dimy-1)/dimy))];
```

There are three parameters in the definition of **“parallel.gpu.CUDAKernel”**, the first parameter is the name of the **ptx** file compiled and created with the CUDA C compiler, the second parameter is the name of the source file containing the CUDA kernel functions, and the third parameter is the parameter that specifies which kernel function to run from within the source file. In the example given below, it is declared to the **kernelMul** kernel function object that the **cudapointProductMatrixGPU** kernel function, which multiplies the elements of two matrices, will be used. In the next step, the data to be calculated using the GPU is sent to the CUDA kernel function from MATLAB as a parameter using the **kernelMul** object, and the data is calculated on the GPU cores. The following script is used to execute the CUDA function by calling a ptx file from MATLAB.

```
im = feval(kernelMul,a,b,c,nx,ny);
```

With the **feval** function in the given script, parameters are passed to the CUDA kernel function using the kernel function object that contains the **ptx** file. The first parameter of the **feval** function is which kernel function object to use, the two and all the following parameters are the formal parameters of the **cudapointProductMatrixGPU** CUDA kernel function.

3. Generating SIM illumination patterns

An application software called **patternGenerator** has been developed for generating SIM illumination patterns. **patternGenerator** takes phase and period values as parameters and creates illumination patterns in binary image format using these values. At least 9 images are needed for SIM reconstruction, and these images are modulated with illumination patterns in different angular orientations and phase values. The sample illumination patterns created for SIM are shown in Figure S4, the function codes developed to create these illumination patterns are given in Figures S5 and S6.

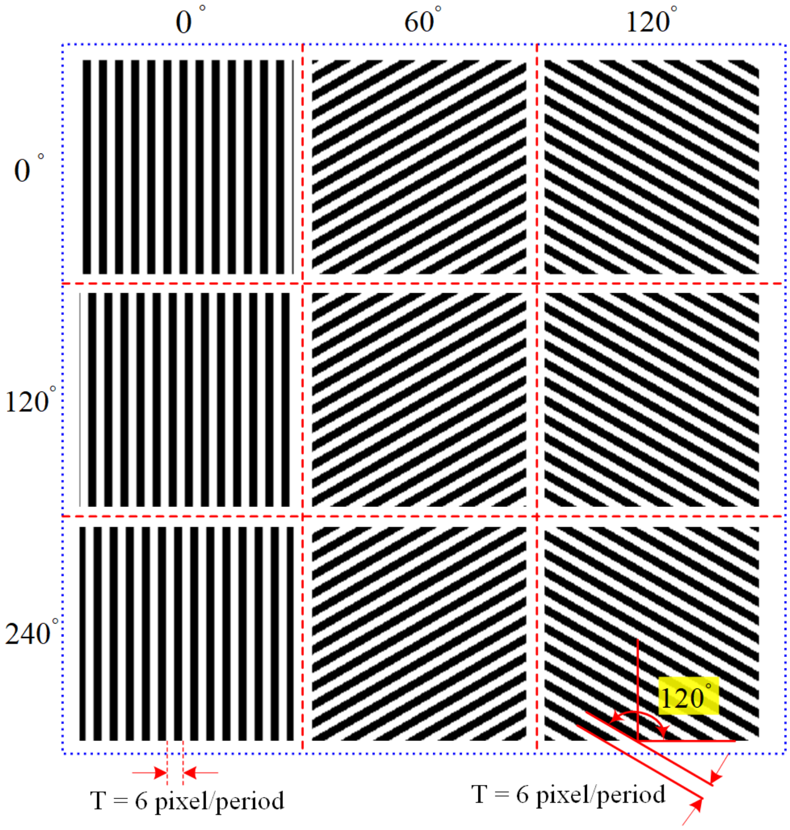

Figure S4. Illumination patterns used for SIM.

```
width  = 684;
height = 608;
im = zeros(width*4,height*4); % DMD mirror array dimension in horizontal and vertica
[row,col] = size(im);
halfperiodPixNum = 3; % represents half period of illumination patterns
i = 1;
j = 1;
k = 1;
rt1 = 42;%60;
% rt1 determines the angular orientation of the illumination pattern,
% the reason being 42 is because each pixel in the DMD chip is magnified 2 time horizontally.
rt2 = -42;%120;
imcounter = 1;
last = halfperiodPixNum;
 while (j < col)
          im(1:end,j:last) = 1;
          j = j + halfperiodPixNum*2;
          last = j;
          last = last + halfperiodPixNum-1;
          if(last > col)
             md = mod(col,halfperiodPixNum);
             if (im(1,col-md) == im(1,col-md+1))
                if (im(1,col-md) == 0)
                   im(1:end,j:col) = 1;
                else
                    im(1:end,j:col) = 0;
                end
             end
             break;
          end
 end
```

Figure S5. MATLAB function developed in order to create illumination patterns in SIM method-part1.

```
pattern = im;
p2 = pattern(1500:2184-1,1000:1608-1);
imwrite(im2bw(p2),['0',num2str(imcounter),'_dlp.bmp'],'bmp');
imcounter = imcounter +1;
% rotate image 60 degrees
rotP = imrotate(pattern,rt1,'nearest','crop');
p =rotP(1500:2184-1,1000:1608-1);
imwrite(im2bw(p),['0',num2str(imcounter),'_dlp.bmp'],'bmp');
imcounter = imcounter + 1;
%rotate image 120 degrees
rotP = imrotate(pattern,rt2,'nearest','crop');
p =rotP(1500:2184-1,1000:1608-1);
imwrite(im2bw(p),['0',num2str(imcounter),'_dlp.bmp'],'bmp');
imcounter = imcounter +1;


shift = 120 / (360/(halfperiodPixNum * 2));
for k = 2:3
    pattern = circshift(pattern,[0  shift]);
    p2 = pattern(1500:2184-1,1000:1608-1);
    imwrite(im2bw(p2),['0',num2str(imcounter),'_dlp.bmp'],'bmp');
    imcounter = imcounter + 1;
    % rotate image 60 degrees
    rotP = imrotate(pattern,rt1,'nearest','crop');
    p =rotP(1500:2184-1,1000:1608-1);
    imwrite(im2bw(p),['0',num2str(imcounter),'_dlp.bmp'],'bmp');
    imcounter = imcounter +1;

    % rotate image 120 degrees
    rotP = imrotate(pattern,rt2,'nearest','crop');
    p =rotP(1500:2184-1,1000:1608-1);
    imwrite(im2bw(p),['0',num2str(imcounter),'_dlp.bmp'],'bmp');
    imcounter = imcounter +1;

end
% A matrix with all values of 1 was created for wide field illumination
% All DMD mirrors will be on when this image is uploaded to DMD
i = ones(684,608);
imwrite(i,'10_dlp.bmp');
```

Figure S6. MATLAB function developed in order to create illumination patterns in SIM method-part2.

4. SIM image reconstruction image pre-processing step

The raw images obtained using the experimental setup created for SIM are combined with the image reconstruction algorithm in order to obtain a super-resolution image. Before processing the raw images with the image reconstruction algorithm, it is necessary to do some pre-processing. It has been observed that there are hot pixels on the obtained raw images due to camera readout noise. Figure S7 shows an exemplary image with such camera readout noise on the raw image.

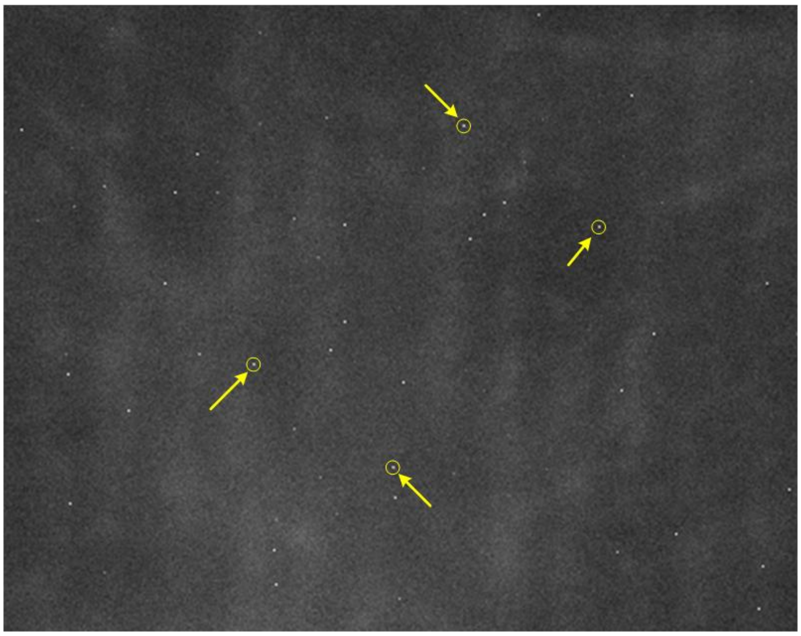

Figure S7. An examplary noisy image caused by camera reading.

Pixels indicated with yellow arrows in Figure S7 are noises caused by camera readout. Median filter is used to clear these noises from the raw image. After the noise caused by the camera readout noise is removed by using the median filter, the pixel density differences of the obtained modulated raw images caused by the camera exposure time or different hardware effects (the DMD LED light source not being at the desired luminance level, etc.) should be eliminated. Each of the obtained raw images can have different brightness and contrast values. For this reason, histogram matching has been done so that all images in the data set have the same contrast value. With histogram matching, each of the raw images is matched to the histogram function distribution of a single image with the highest mean light intensity value of one of the images in the data set.

5. Estimation of Experimental Parameters

Calculation of illumination pattern phase shift in image reconstruction

A Gaussian high-pass filter is applied to estimate the illumination pattern phase shift of raw images modulated for SIM reconstruction. The formula for the Gaussian high-pass filter is given as:

$$H(u,v) = 1 - e^{-\frac{D^2(u,v)}{2D_0^2}} \qquad \text{(S1)}$$
$$D(u,v) = [(u - M/2)^2 + (v - N/2)^2]^{1/2}$$

where D_0 is the sigma value and it has been chosen to be as smaller than the OTF cutoff frequency. In Figure S8, the filtering step of the 0° angular orientation image obtained with the Gaussian high-pass filter is shown.

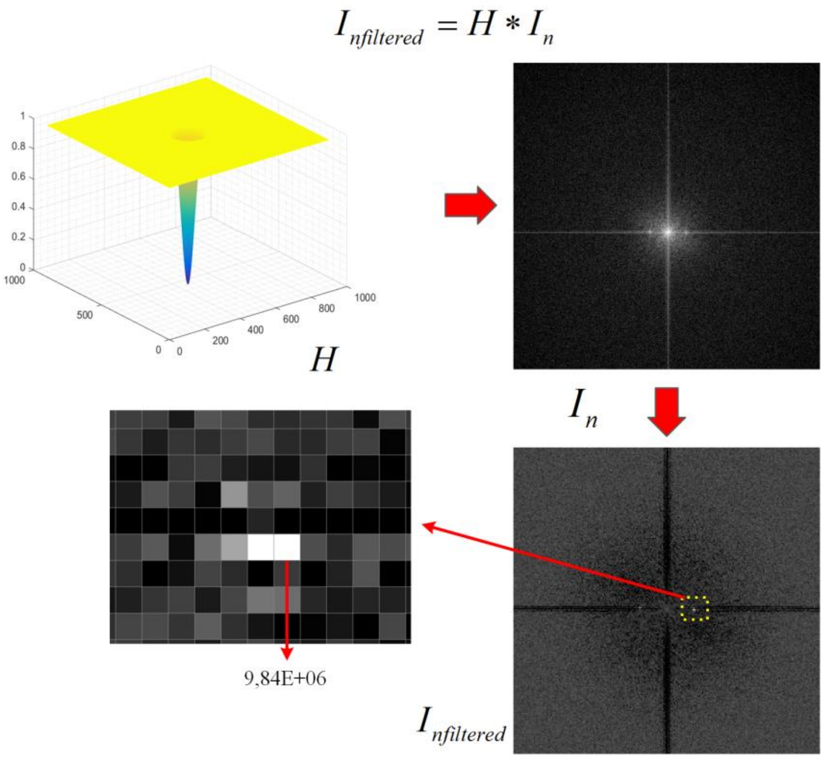

Figure S8. Image with 0° angular orientation filtered with a Gaussian high-pass filter.

Calculation of illumination pattern spatial frequency in image reconstruction

First, a low-pass filter is applied to each acquired image to suppress high frequency information outside the OTF cutoff frequency in order to calculate the spatial frequency of the illumination pattern. The low-pass filter applied to suppress the high frequency information outside the OTF cutoff frequency is given as:

$$\widetilde{D}_n filtered(k) = \frac{(OTF)^* \cdot \widetilde{D}_n(k)}{|OTF| + \sigma} \qquad \text{(S2)}$$

where, OTF is the optical transfer function, $\widetilde{D}_n$ is the frequency domain equivalent of the resulting image, and σ is a small positive constant that ensures no division by zero error and fine tuning of noise. In the next step, the ideal high-pass filter is applied to eliminate the DC component. Finally, the Equation S3 was used to calculate the difference

between the absolute value of the modulated image's frequency response and the absolute value of the image obtained with wide field illumination.

$$\widetilde{DF}_n(k) = \left|\widetilde{D}_n(k)\right| - 3 \cdot \left|\widetilde{W}\right| \tag{S3}$$

In this way, the dominance of the frequency information of the illumination patterns in the images obtained by SIM has been increased. In Equation S3, the factor of 3 in the absolute value of the wide-field illumination image was determined as a result of the experiments.

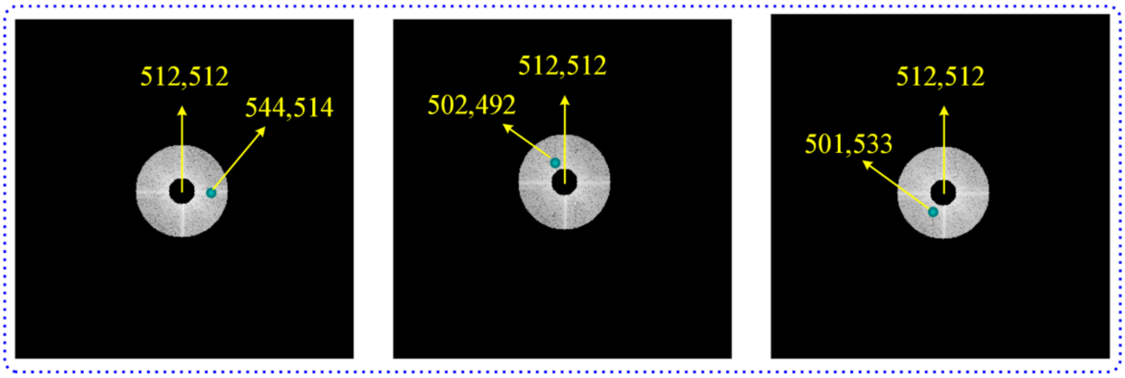

Figure S9. Calculation results of the positions of the spatial frequency vector of the illumination pattern in three different angular orientations.

The positions of the frequency components determined as a result of the calculations of the spatial frequency vector of the illumination pattern are shown in Figure S9. The distance from the center of the frequency component in all angular orientations is recorded as the illumination pattern k_{θ_n} (where k_{θ_n} denotes the amount of shift of the separated frequency components).

Figure S10 shows the developed sim image reconstruction algorithm pseudocode.

Algorithm 1 LED-Based Structured Illumination Microscope Image Reconstruction Algırithm

/ Number of input raw images N = 3, M = 3 */*
/ Raw images filtering, k = 9 */*
for $i = 1, 2, \ldots, k$ **do**
    $f_i(x, y) = median\{g_i(x, y)\}$
end for

/ Calculation of fourier transform of dn raw images $D_{n,m}(r)$, n = orientation, m = phase value of raw images*/*
for $n = 1, 2, \ldots, N$ **do**
    for $m = 1, 2, \ldots, M$ **do**
        $\tilde{D}_{n,m}(k) = \mathcal{F}\{D_{n,m}(r)\}$
    end for
end for

/ Phase shift and illumination frequency estimation of the modulated images*/*
for $n = 1, 2, \ldots, N$ **do**
    for $m = 1, 2, \ldots, M$ **do**
        $P_{n,m} =$ **calculatePhaseShift**$(\tilde{D}_{n,m}(k))$
        $F_{n,m} =$ **calculateIlluminationFrequency**$(\tilde{D}_{n,m}(k))$
    end for
end for

/ Separation of frequency components and obtaining separated frequency components, $m = 1 \rightarrow \tilde{S}_1(k)$, $m = 2 \rightarrow \tilde{S}_2(k + p_\theta)$, $m = 3 \rightarrow \tilde{S}_3(k - p_\theta)$, Shifting the separated frequency components to their original positions*/*
for $n = 1, 2, \ldots, N$ **do**
    for $m = 1, 2, \ldots, M$ **do**
        $\tilde{S}_{n,m}(k) =$ **separateFrequencyComponents**$(P_{n,m}, \tilde{D}_{n,m}(k))$
        $\tilde{S}_{shifted \rightarrow n,m}(k) =$ **shiftSeparatedComponents**$(F_{n,m}, \tilde{S}_{n,m}(\mathbf{k}))$
    end for
end for

Combining the separated components $\tilde{S}_{shifted \rightarrow n,m}(\mathrm{k})$ shifted to their original positions using the wiener filter, combined SIM images $\tilde{S}_{SIM}(k)$.

return $\mathcal{F}^{-1}\{\tilde{S}_{SIM}(k)\}$

Figure S10. Structured illumination microscopy image reconstruction algorithm pseudocode

6. SIM Reconstruction with Artificial Image

In this section, in order to ensure the validation of the SIM image reconstruction algorithm, a test was performed using raw images obtained by modulating an artificial image. Images modulated with illumination patterns created with artificial images, as well as their frequency spectrums, can be observed in Figure S11.

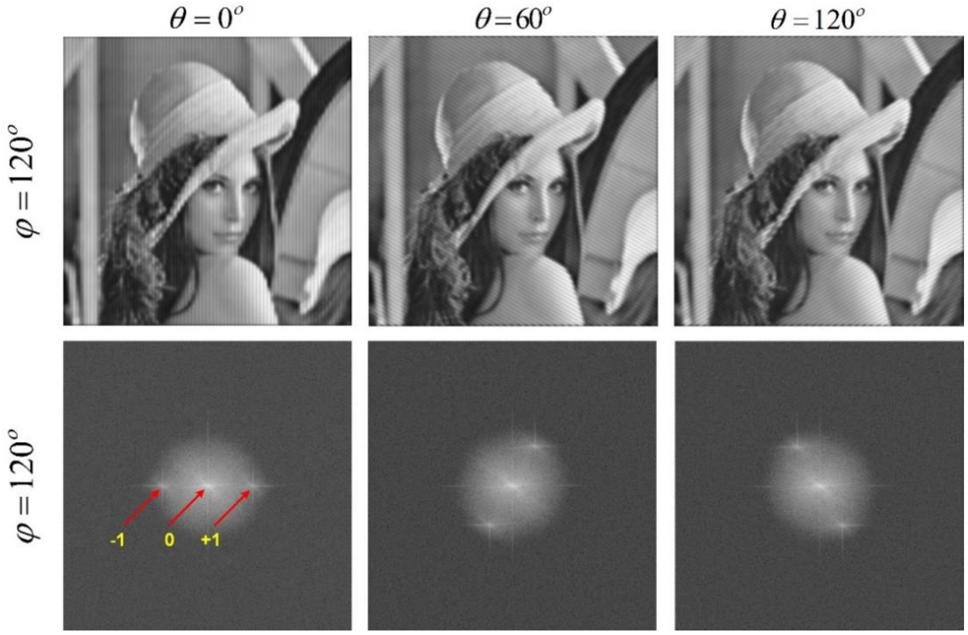

Figure S11. The frequency spectrums of images modulated with illumination patterns of three different orientations and the same phase value.

The developed SIM reconstruction algorithm was initially tested on an artificial image, and the algorithm's accuracy was validated. The result of SIM reconstruction using artificial images is shown in Figure S12.

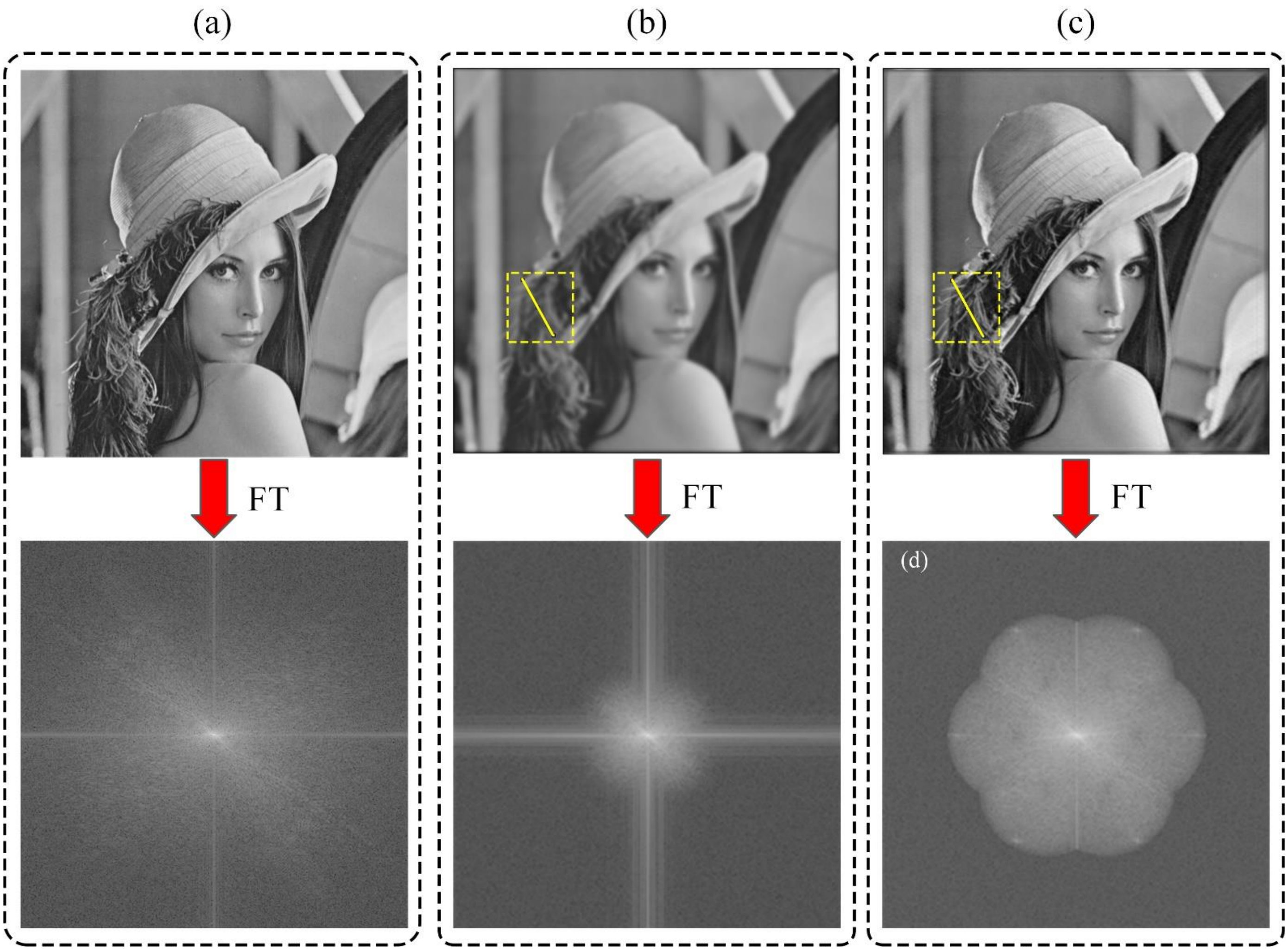

Figure S12. Wide field and sim images obtained as a result of sim reconstruction, (a) original image and its frequency spectrum, (b) wide-field image and its corresponding OTF-suppressed frequency spectrum, (c) Reconstructed SIM image and its corresponding OTF cutoff extended frequency spectrum

In the experiment study with artificial image, wide field image was created as a result of convolution operation of the artificial image with a psf in frequency space to indicate the psf effect of the microscope. Line intensity profile of the region marked in the square area defined by yellow dashed lines in Figure S12(a,b) is shown in Figure S13.

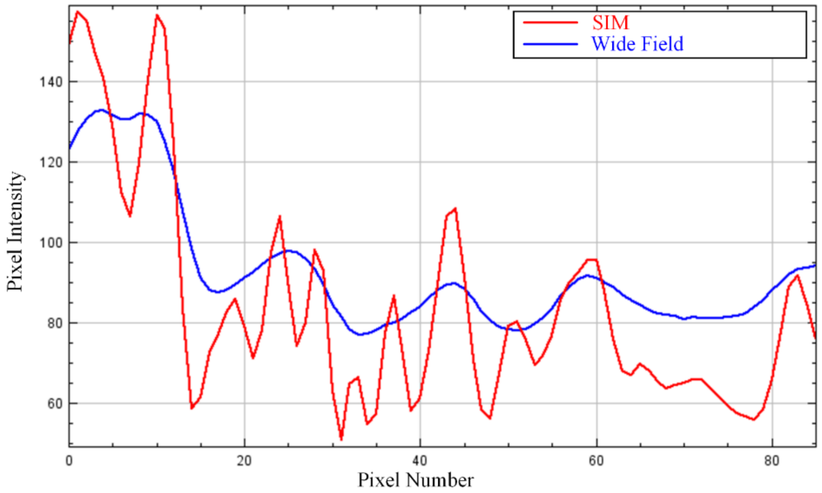

Figure S13. Pixel values change graph as a result of image reconstruction operation

7. Additional SIM reconstruction results

Figures S14 and S15 show additional SIM reconstruction results. When compared with the images recorded using wide-field illumination, improvements in signal to noise ratio and resolution due to SIM reconstruction are clearly visible in these figures.

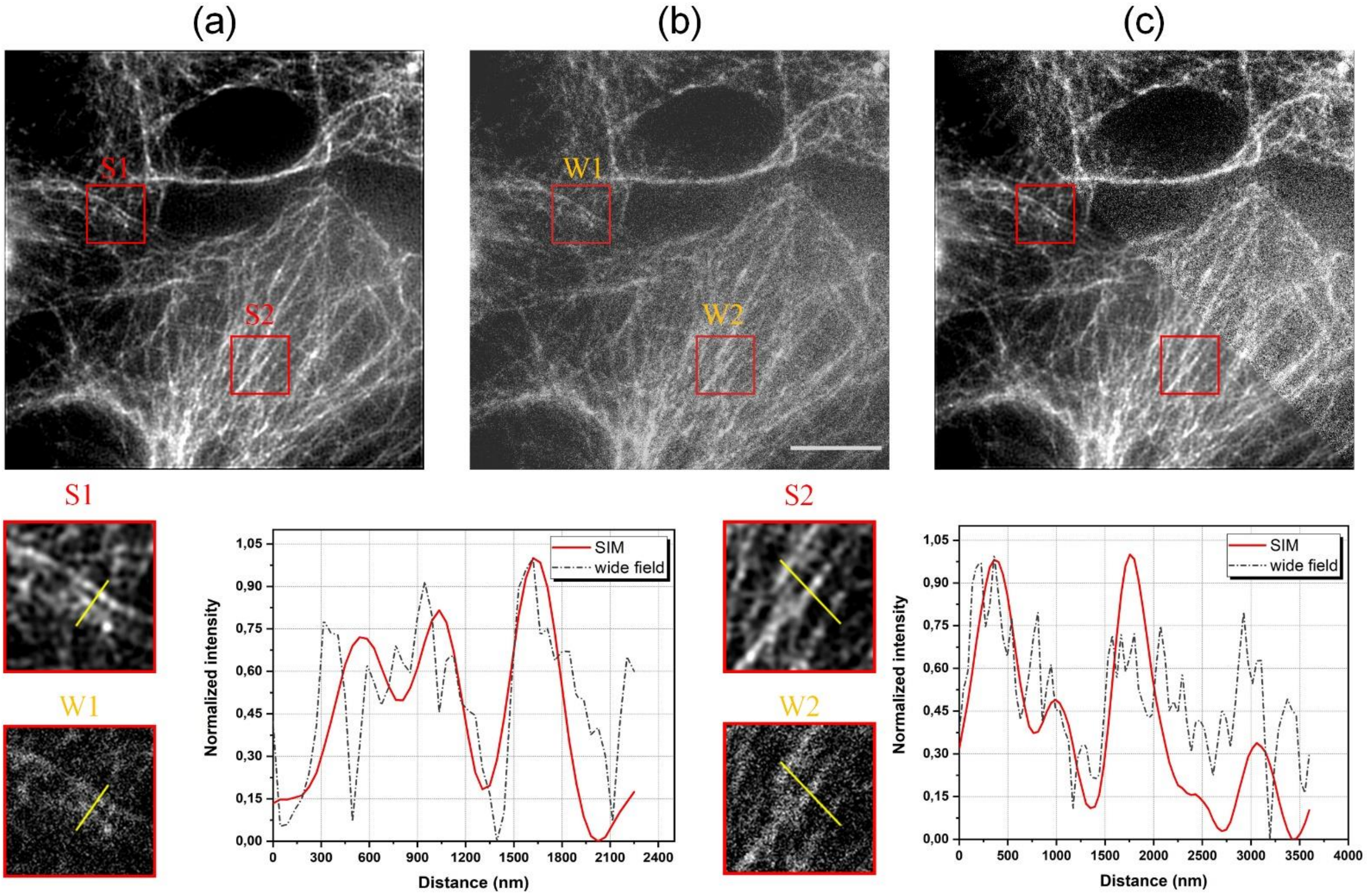

Figure S14. (a) High resolution image obtained as a result of SIM image reconstruction (S1 and S2 indicates ROIs of reconstructed SIM images, respectively), (b) image obtained with wide field illumination (W1 and W2 indicates ROIs of reconstructed wide field illumination images, respectively), (c) combined SIM and wide field illumination images, (b) scale bar indicates 10 *µm.*

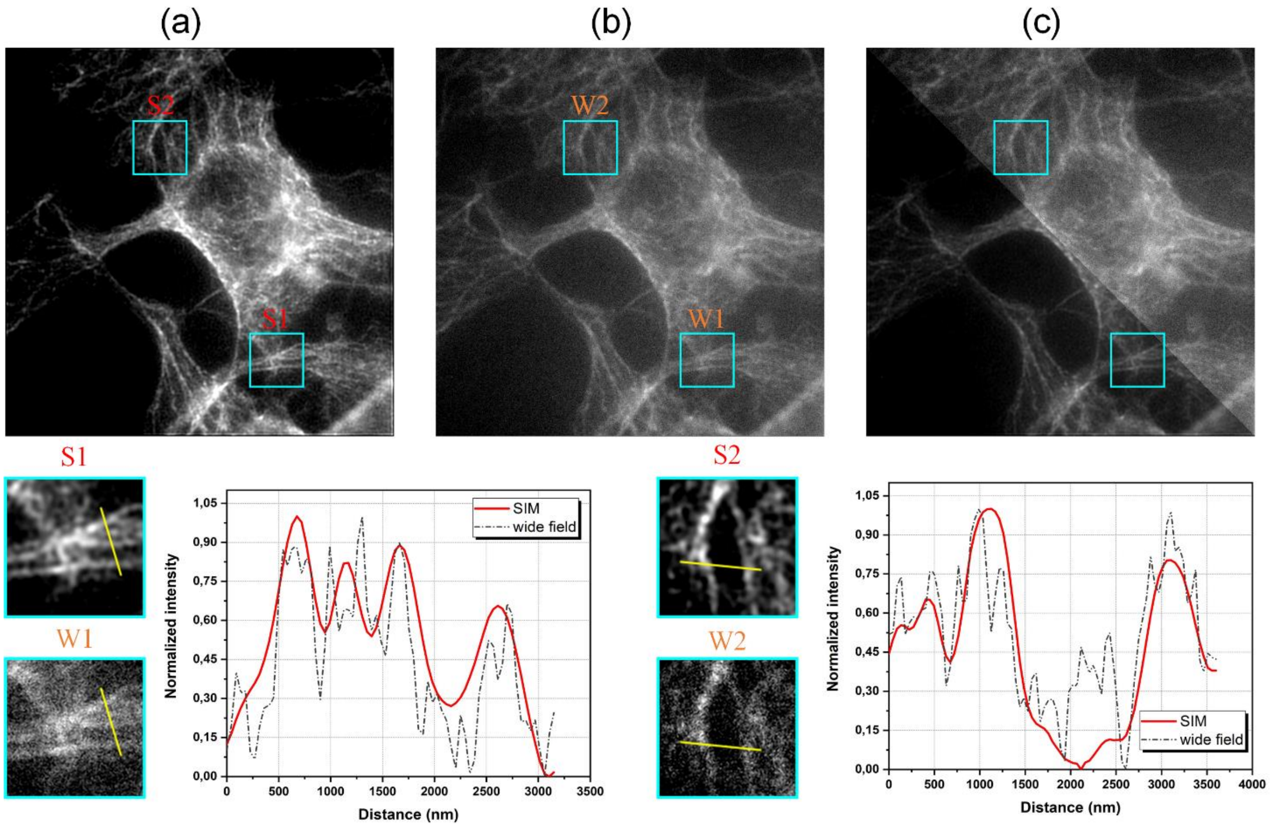

Figure S15. (a) High resolution image obtained as a result of SIM image reconstruction (S1 and S2 indicates ROIs of reconstructed SIM images, respectively), (b) image obtained with wide field illumination (W1 and W2 indicates ROIs of reconstructed wide field illumination images, respectively), (c) combined SIM and wide field illumination images, (b) scale bar indicates 10 *μm.*

8. Picture of the experimental setup

Figure S16 shows a picture of the experimental setup.

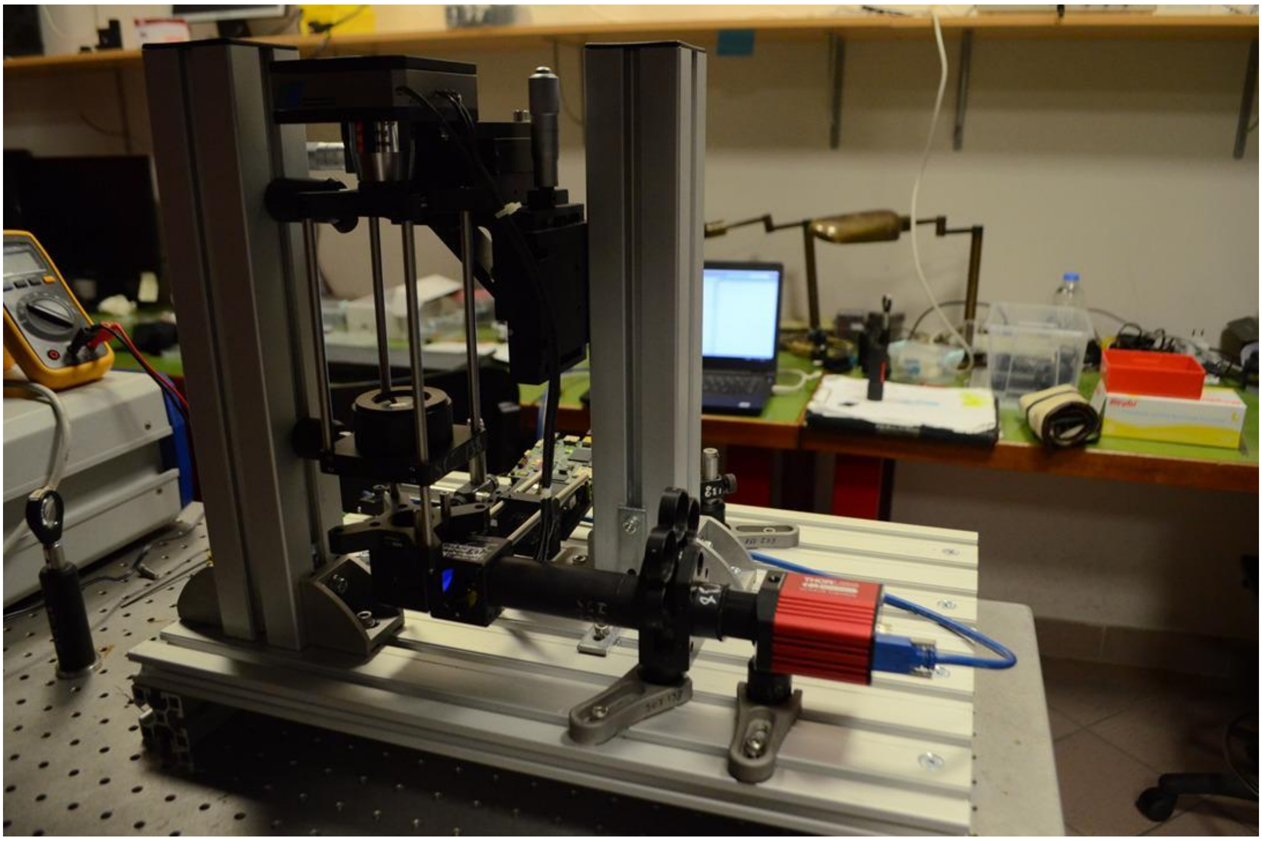

Figure S16. LED illuminated structured illumination microscopy experimental setup.

9. Materials

COS-7 (African green monkey kidney cells, CRL-1651; ATCC)

Dulbecco's modified Eagle's Medium DMEM/F12 50/50 medium (Pan Biotech, Vienna, AUT)

Fetal Bovine Serum (FBS, Life Technologies, Carlsbad, Ca, USA)

Penicillin-streptomycin (Gibco, Thermo Fisher Scientific)

MycoAlert Mycoplasma Detection Kit (Lonza, Basel, CH)

BSA (Cat. # BSA-1T, Capricorn Scientific, Ebsdorfergrund, DE)

Primary antibody: mouse anti alpha-tubulin (Sigma, DM1A)

Secondary antibody: AlexaFluor 488-coupled (Life Technologies)

Mowiol mounting medium containing N-propyl gallate (Sigma-Aldrich)

Cell Culture

COS-7 (African green monkey kidney cells, CRL-1651; ATCC) were cultured with Dulbecco's modified Eagle's Medium DMEM/F12 50/50 medium (Pan Biotech, Vienna, AUT) supplemented with 10% Fetal Bovine Serum (FBS, Life Technologies, Carlsbad, Ca, USA) and 1% penicillin-streptomycin (Gibco, Thermo Fisher Scientific) with %5 CO_2 in 37°C. Cell line was tested for mycoplasma by MycoAlert Mycoplasma Detection Kit (Lonza, Basel, CH).

Immunoflourescence

Cos-7 cells were grown on coverslips to reach 80-90% confluency, washed twice with PBS and fixed in ice cold methanol at -20°C for 10 minutes. Following rehydration in PBS, cells were blocked with 3% BSA (Cat. # BSA-1T, Capricorn Scientific, Ebsdorfergrund, DE) in PBS + 0.1% Triton X-100 followed by incubation with primary antibody mouse anti alpha-tubulin (Sigma, DM1A) at 1:10.000 in blocking solution for 1 hour at room temperature. Cells were washed three times with PBS and incubated with secondary antibody AlexaFluor 488-coupled (Life Technologies) at 1:2000 in blocking solution for 1 hour at room temperature. Following three washes with PBS, cells were mounted using Mowiol mounting medium containing N-propyl gallate (Sigma-Aldrich).
